# Transmission on empirical dynamic contact networks is influenced by data processing decisions

**DOI:** 10.1016/j.epidem.2018.08.003

**Published:** 2018-08-29

**Authors:** Daniel E. Dawson, Trevor S. Farthing, Michael W. Sanderson, Cristina Lanzas

**Affiliations:** a Department of Pathobiology and Population Health, College of Veterinary Medicine, North Carolina State University, Raleigh, NC, 27606, USA; b Center for Outcomes Research and Epidemiology, Department of Diagnostic Medicine and Pathobiology, College of Veterinary Medicine, Kansas State University, Manhattan, KS, USA

**Keywords:** Dynamic contact data, Data processing, Entropy, Contact networks, Transmission dynamics, Disease transmission model

## Abstract

Dynamic contact data can be used to inform disease transmission models, providing insight into the dynamics of infectious diseases. Such data often requires extensive processing for use in models or analysis. Therefore, processing decisions can potentially influence the topology of the contact network and the simulated disease transmission dynamics on the network. In this study, we examine how four processing decisions, including temporal sampling window (TSW), spatial threshold of contact (SpTh), minimum contact duration (MCD), and temporal aggregation (daily or hourly) influence the information content of contact data (indicated by changes in entropy) as well as disease transmission model dynamics. We found that changes made to information content by processing decisions translated to significant impacts to the transmission dynamics of disease models using the contact data. In particular, we found that SpTh had the largest independent influence on information content, and that some output metrics (R_0_, time to peak infection) were more sensitive to changes in information than others (epidemic extent). These findings suggest that insights gained from transmission modeling using dynamic contact data can be influenced by processing decisions alone, emphasizing the need to carefully consideration them prior to using contact-based models to conduct analyses, compare different datasets, or inform policy decisions.

## 1. Introduction

The nature and intensity of the type of contact that facilitates disease transmission is one of the defining features of the epidemiology of infectious diseases and inherently influence disease dynamics in populations as well as efforts aimed at control. Contact variation is one of the most important sources of transmission heterogeneity (Bansal et al., 2007). However, mathematical models have often assumed homogeneous contact patterns in which individuals have the same number of random contacts (Begon et al., 2002). The incorporation of realistic heterogeneous contact structures into disease transmission models alters infection dynamics relative to when homogeneous contact structure is assumed (Bansal et al., 2007; Duncan et al., 2012), and has provide insights into disease dynamics historically obscured by omission of contact data (Craft, 2015). Thus, using contact structure in disease models can contribute to greater model realism and potentially better model applicability (Masuda and Holme, 2013). In addition to disease models, network theory has also been applied to social contact data to identify network features that are associated to particular patterns of disease spread (Christley et al., 2005; Cross et al., 2012; Hamede et al., 2009).

Most of the work incorporating contact structures in disease modeling uses static networks in which the interactions among individuals remain constant over time (Holme, 2013; Keeling and Eames, 2005). More recently, empirical contact data has become available to model disease transmission on dynamic networks of contacts, thus providing insights on how contact timing and variation overtime influence disease dynamics (Barrat et al., 2014; Karsai et al., 2011; Masuda and Holme, 2013). In particular, telemetric technologies have facilitated the collection of empirical contact data at a very high spatial and temporal resolution (Chen et al., 2014a; Craft, 2015; Duncan et al., 2012), which we refer here as dynamic contact data. Dynamic contact data are generally collected in two ways: with proximity sensors or radio telemetry (RT) within a sensor field. With proximity sensors, individuals are outfitted with devices that detect other devices within a particular proximity and record durations of contacts between individuals (Barrat et al., 2014; Isella et al., 2011; Stehlé et al., 2011). In RT systems, individuals are outfitted with devices that transmit a radio signal to be received by sensors that provide information on locations of individuals over time (Böhm et al., 2018; Chen et al., 2014b; Perkins et al., 2009).Then, contact structure is inferred by temporally aligning the locations of individuals and applying a proximity threshold of contact.

Insights on transmission gained from telemetric technologies rely upon the assumption that the record of empirical contacts reflects potential transmission events (Eames et al., 2015). However, processing dynamic contact data in preparation for analysis can itself influence the resulting structure of the data (Eames et al., 2015; Haddadi et al., 2011), making disease transmission in a model more or less likely to occur. In particular, in situations where there are shared resources such as discrete food or water locations, short, frequent contacts between individuals during which transmission may not occur (i.e., “non-effective contacts”) may inflate and obscure effective contacts for transmission (Psorakis et al., 2012; Spiegel et al., 2016). On the other hand, if contact criteria are too stringent, effective contacts can be under predicted. Despite the importance of processing decisions on defining the contact structure (Craft, 2015), few studies have explicitly considered their influence (Haddadi et al., 2011). Furthermore, processing decisions are often not explicitly explained or justified.

Here, we consider four types of dynamic contact data processing decisions that may influence contact and transmission dynamics in RT-based data: 1) temporal sampling window (TSW), 2) the spatial threshold for contact (SpTh), 3) the minimum duration of contact (MCD), and 4) scale of temporal aggregation. Table 1 provides a graphical explanation of each processing decision and its potential implications for disease transmission. In brief, data is first temporally aligned to determine if individuals shared spatial locations contemporaneously into TSWs of the same length (e.g. 10 s). An individual’s location during the window is determined by taking an average of recorded locations during the specified time length (Haddadi et al., 2011). The SpTh refers to the proximity at which an effective contact is considered possible, and is obtained by calculating distances between individuals at each TSW (e.g., with Euclidean methods (Chen et al., 2013)), then applying the SpTh as a filter. The MCD is the minimum number of sequential TSWs in which the SpTh has been breached before pathogen transmission is possible. Lastly, fully processed dynamic contact data (i.e. TSW, SpTh, and MCD have been applied) are often aggregated into proximity-based social networks (Spiegel et al., 2016) at specific temporal scales (e.g. hours or days) to characterize contact structure or disease transmission functions prior to use in transmission models.

The objective of our study was to evaluate the effect of processing RT data on the information content of the data and simulated disease dynamics. For that purpose, we processed a set of dynamic contact data of cattle locations in a feedlot pen, collected via RT methods, varying the TSW, the SpTh, the MDC, and temporal aggregation in a factorial manner. We then adapt and expand upon previously suggested methods (Haddadi et al., 2011) that calculate changes in information content of datasets, as indicated by graph entropy, to quantify the effect of processing decisions on information content of the data. Next, we explicitly examine how decisions influence transmission dynamics by using the processed datasets in a network-based simulation model of transmission and relate changes in information content to changes in disease dynamics. Lastly, we propose a generalized methodology to determine processing criteria for the processing decisions investigated.

## 2. Methods

### 2.1. Data acquisition

Positional data were collected from research facilities affiliated with the Kansas State University College of Veterinary Medicine. Data consisted of locations of 70 beef cattle calves within an approximately rectangular feedlot pen (28 m^2^ in area per calf) continuously monitored from May 5th to May 25th, 2016 (21 days). Each calf was labeled with a radio transmitting ear tag (Smartbow, Austria) designed to transmit a signal to receivers positioned around the pen, and had a maximum temporal resolution of 5–10 s. System software then used triangulation methods to calculate x–y position for observation and logged the position data to a central server.

### 2.2. Data processing

**Fig. 1.**
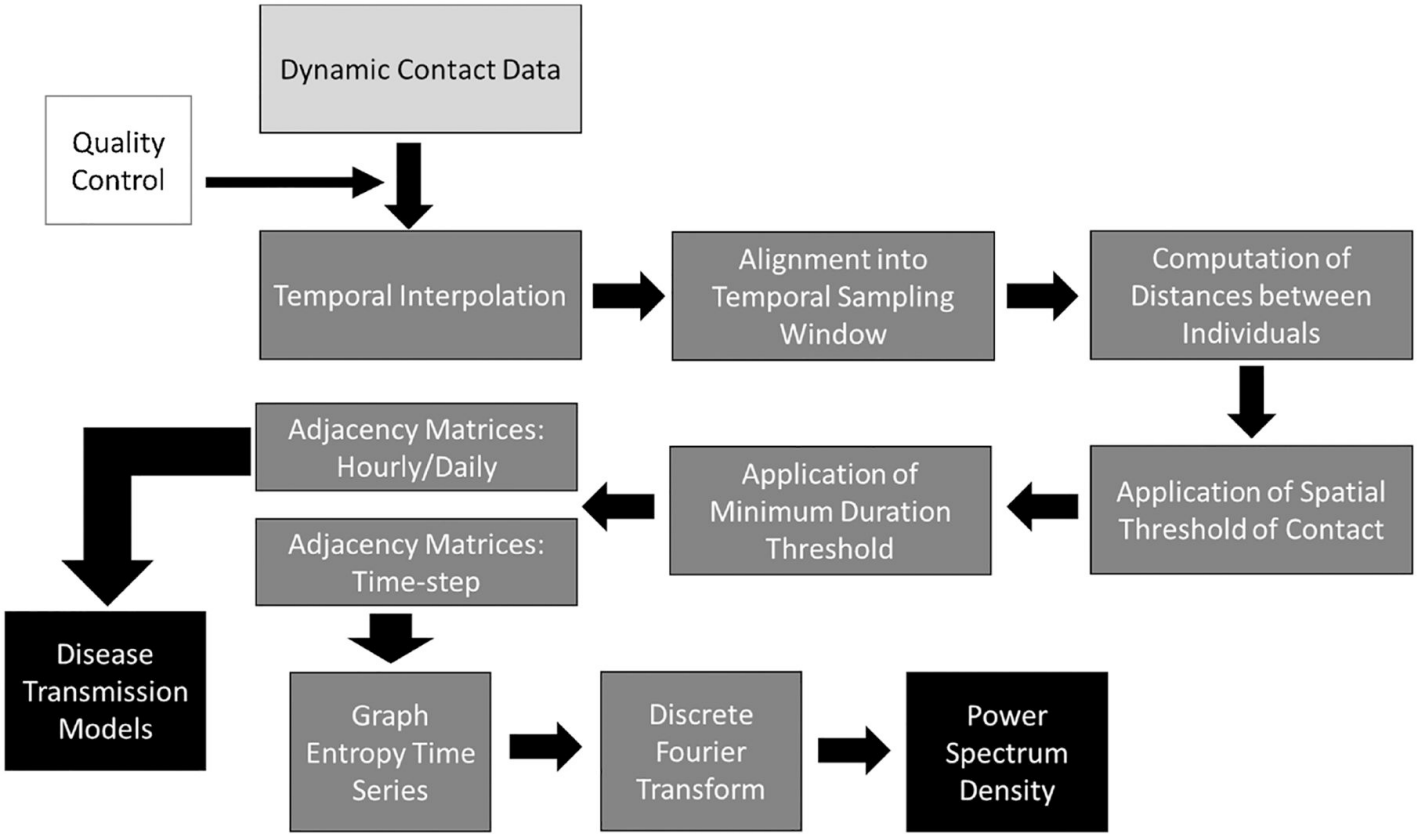
Flow chart of data processing procedures. Flow chart components include unprocessed temporally dynamic contact data (light grey), processes and intermediate products (dark grey), final products (black), and quality control processes (white).

Prior to processing, raw locational data underwent several pre-processing procedures for quality control purposes. See Supplemental Materials (S1.1) for further details of these procedures. After pre-processing, data processing consisted of four main parts: temporal interpolation, averaging data into consecutive temporal windows (TSW), contact estimation at particular spatial thresholds (SpTh), and restriction of contact records to those meeting minimum contact duration criteria necessary for disease transmission (MCD) (see Fig. 1 for an overview). For a detailed account of each step, see Supplemental Materials (S1.2). In addition, R-scripts that process generalized spatiotemporal contact data (i.e., spatial locations and timestamps) for individual animals are supplied in the Supplemental Materials (S2). In brief, discontinuous locational data (received every 5–10 s) were used to interpolate a value to every second of the dataset, creating a continuous temporal record for each day of the dataset. Then, these values were averaged into TSWs. Next, distances between cattle at each temporal window was calculated, followed by the application of first a SpTh, and then a MCD to determine whether a contact occurred in a given TSW. After these steps, the processed data was routed in the form of adjacency matrices towards analyses of how either contact information or disease transmission dynamics change with processing decisions.

### 2.3. Contact data information loss

We characterized the amount of information loss resulting from processing methodologies by quantifying the entropy of dynamic contact data, as suggested by Haddadi et al (2011). Entropy is a measure of the uncertainty or potential information associated with a value of a random variable (Stone, 2015). In the context of networks, the entropy rate characterizes the ease of information flow (e.g. disease transmission) and uncertainty of movement path as it diffuses via contacts through the network (Gómez-Gardeñes and Latora, 2008). Larger entropy rates represent greater efficiency of diffusion and therefore greater uncertainty as to which paths information may take through a network (Gómez-Gardeñes and Latora, 2008; Sinatra et al., 2011). Our approach to calculate information loss included three steps. First we calculated entropy for each TSW. The resultant time series of entropy values were then converted from the time scale to the frequency scale and an average spectral density was calculated (per unit of temporal aggregation(hour or day)). Finally, we calculated the percent of frequency content relative to the maximum. These procedures follow those proposed by Haddadi et al. (2011), but were applied to each factorial combination of data processing decisions considered, and were modified to weight TSW-based contacts (i.e., contact = 1 or 0) by the number of sequential contact durations where contacts occur (i.e., contact = 1).

In the first step, we calculated the entropy rate for each adjacency matrix (*a*) at each TSW-based time step, creating a time series of entropy values for each generated dynamic contact data. Time series ranged in length from 181,440 steps, when TSW was 10 s, to 10,080 steps, when TSW was 180 s. The entropy rate (h) for a graph is the sum of contributions from each node in the network, and is calculated as:

(1) $$h=-\sum_{i,j,k}\pi_{ji}\times w_{ik}ln\left(\pi_{ji}\right)$$

where π_*ji*_ is a transition matrix of *a*_*ij*_ of a random walk (a random walk Laplacian (Haddadi et al., 2011)), and *w* is the stationary distribution of the Markov chain of a random walk started in each connected graph *k* of π_*ji*_. The transition matrix (π_*ji*_) was calculated similarly as described in (Gómez-Gardeñes and Latora, 2008):

(2) $$\pi_{ji}=\frac{a_{ij}f_{ij}}{\Sigma_{j}a_{ij}f_{ij}}$$

with *a*_*ij*_ representing a contact on the adjacency matrix (a_ij_ = 1 if contact, 0 if no contact) between node *i* and node *j*, and *f*_*ij*_ was the number of contact durations in which the time-step based contact between *i* and *j* was currently involved. For example, if a single time-step based contact (i.e., 1) between node *i* and *j* was part of a two-sequential-TSW duration contact, then *f*_*ij*_ = 2. *f*_*ij*_ was incorporated to account for the fact that many contacts persisted across time steps. Contacts with longer durations could bias a random walk, reducing entropy. Isolated nodes were assigned a value of 1 where *i* = *j,* such that each column summed to 1. In each connected subgraph, there is a stationary Markov distribution *w*_*k*_ representing the stationary distribution of long-term probabilities of a biased random walker being on the nodes of the graph if a random walk is initiated at any particular node in the subgraph *k* (Gómez-Gardeñes and Latora, 2008). To calculate w_ik_ values, connected subgraphs (*k*) greater than size 2 were identified in the overall graph (isolated nodes and subgraphs of length 2 contribute no entropy), and an index vector representing the start of a random walk at a particular node in *k* was iteratively multiplied against π (1000 times was shown to be sufficient during method development) to produce w_k_. Some subgraphs existed as linear chains or non-triad cycle graphs, causing the stable Markov chain to oscillate between two sets of probabilities. In these cases, the two sets of distinct probabilities over 1000 iterations were weighted by 0.5 (the relative probability of each set occurring during a random walk), and combined to construct w_k_. Lastly, *h* for a graph was computed by summing the individual entropy values contributed from each subgraph using the appropriate w_k._ The result of this were time series of h values over the total 21-day length for each set (n = 80) of processed data.

To condense graph information for comparison over the entire temporal span of contact data, entropy time series were converted to the frequency domain by applying the Fast Fourier Transform, producing the discrete Fourier transform of each time series. Then, each Fourier transform was summarized by the calculation of its power spectral density (Stoica and Moses, 2004):

(3) $$Power\;Spectrum\;Density=\sum_{h=1}^{N}1/N|X[h]{|}^{2}$$

in which N = total samples in set, h = entropy time series, and *X*[*h*] is the Discrete Fourier Transform of h. The power spectrum density (or simply, spectral density) of a Fourier transform is a single value that represents the total power of a signal averaged across its component frequencies (Stoica and Moses, 2004), and thus serves as reasonable proxy of information content captured by the time series of entropy values in a dataset. Fourier transform operations, as well as pre-(de-trending and tapering entropy values prior to Fourier transform) and post-(smoothing frequency spectra via a Daniell kernel) processing steps, were carried out using the *spectrum* function in the stats package (R Core Team, 2017).

Upon conversion to the frequency scale, we compared the effect of the different processing decisions (SpTh, TSW and MCD) on information content at the hourly and daily scales as follows. As an initial parsing methodology, we first conducted partial ranked correlation coefficient analyses (PRCC) to determine how the three parameters individually contributed towards spectral density considered at hour and day scales (Marino et al., 2008; Wu et al., 2013). This analysis showed that SpTh was the most influential independent parameter of the three, so spectral densities of factorial datasets were considered based on bins of SpTh (0.1665, 0.333, 0.666, 0.999 m). Within SpTh bins, the average percentage of the maximum spectral density per hour or day in each dataset (always the dataset with highest information content, i.e. TSW = 10 s and MCD = 1) was calculated. Percentages of maximum spectral density were also visualized over spans of 24 h and 21 days, respectively to allow for daily and hourly patterns in information change to be ascertained. An R-script that performs the above procedures on a generalized, processed contact dataset is supplied in the Supplemental Materials (S3). Lastly, to provide a point of comparison with the entropy-based metric considered here, two typical metrics of contact (average per capita contact durations, average per capita contacts) were calculated on an hourly and daily basis.

### 2.4. Pathogen transmission dynamics

The influence of SpTh, TSW and MCD on potential transmission dynamics was investigated by simulating transmission of a organism similar to the enteric pathogen *Escherichia coli* 0157 on a network-based Susceptible-Exposed-Infected-Recovered type transmission model. Though not pathogenic in cattle, *E. coli* 0157 is an important pathogen of humans that is commonly reported in cattle herds in various production systems (Ekong et al., 2015; Hussein, 2007; Hussein and Bollinger, 2005). To compare the influence of temporal aggregation, the model was simulated using a time series of adjacency matrices aggregated at either the hourly (504 h) or daily (21 days) scale for each parameter combination (n = 80). Individual cattle could be in one of four epidemiology states including susceptible, exposed (infected but latent), infectious, and recovered. Although partial immunity in cattle is more likely (Peterson et al., 2007; Thornton et al., 2009), for simplicity we assumed 100% immunity with recovery. Infectious individuals had a level of infectiousness represented by the colony forming units (CFU) (See Supplemental Materials: S4) associated with a contact. Dose received by one individual through contacts with infectious individuals were accumulated and evaluated at each hour or day. The probability of colonization was a dose-dependent function:

(4) $$pcol=\frac{1}{1+K/CFU_{cum}}$$

where CFU_cum_ is the dose accumulated over the evaluation period (hour, or day) contributed by infectious individuals. CFU_cum_ was calculated as the cumulative duration of contact periods with infectious individuals (of length of the TSW) * *CFU per contact duration (*CFUpcd*)*. K was the dose that yields a 50% probability of colonization (Gadagkar and Call, 2015). A random sample drawn from a Bernoulli distribution (1, p = *pcol*) determined transmission success or failure. When multiple infectious individuals contributed CFU’s to cause a single infection, the individual contributing the most CFU’s was selected as the infecting individual. Successfully colonized individuals were transferred to exposed status, while non-successfully colonized individuals remained as susceptible. All contacts between infectious individuals and susceptible ones were assumed temporally independent. The evaluation of the function over a time interval used only the accumulated CFUs transferred during contact with infectious individuals over that interval. Thus, cattle did not have a memory of CFU’s accumulated prior to the current evaluation period (hour or day). Colonized individuals were transferred to recovered status after their time to recovery (i.e., their infectious period) had elapsed, and they were no longer infectious. All simulations started with a single, randomly selected infectious individual, and ran through either the entire time series, or until no infectious individual remained.

Constant model parameters included the *time of latency* (24 h), *time to recovery* (240 h), *K* (10,000 CFUs), and the *CFUpcd* (100 CFUs). The first three model parameter values were based on those reported for *E. coli* 0157 (Besser et al., 2001; Cray and Moon, 1995; Kulow et al., 2012; McGee et al., 2004). The value of *K* was within the range of inoculation doses for *E. coli* O157 reported to be infectious in cattle (Besser et al., 2001; Cray and Moon, 1995). The *CFU*pcd was determined through preliminary analysis to be sufficiently high to reduce the influence of highly connected individuals on subsequent transmission dynamics (S4.1), and was within a range at which transmission-dynamics metrics were relatively insensitive to it (S4.2). A more detailed description of the process used to determine *CFUpcd* and an R-script that simulates disease transmission over a temporal sequence of contact networks is supplied in the Supplemental Materials (S4,5).

#### 2.4.1. Transmission model analysis

The influence of processing decisions on transmission dynamics was considered for three simulation metrics, including R_0_ (the total number of new cases caused by the initially infected individual during its infectious period in an otherwise susceptible population), the time (in hours) to peak infection, and the number of total cattle infected at the end of the simulation. To relate these outputs to information content, output values were averaged over all simulation replicates of each combination of processing criteria, and then plotted against log-transformed spectral density. Lastly, to understand how individual processing criteria related to each metric, separate statistical models were constructed for each response variable using temporal aggregation (daily or hourly), SpTh, TSW, and MCD as predictor variables. See Supporting Information (S8) for details of these models.

## 3. Results

### 3.1. Contact data information content

SpTh was the most influential processing criteria in determining the amount of spectral content of contact data, followed by TSW and MCD for data aggregated both by both hour and day (Table 2). The PRCC for SpTh was strongly positive, consistent with the fact that more contact information is gathered as the SpTh around an individual increases. The PRCCs for TSW and MCD were negatively correlated with spectral content, consistent with the fact that information decreases as the opportunity for contacts decreases due to either reduced sampling, or more stringent contact criteria.

**Fig. 2.**
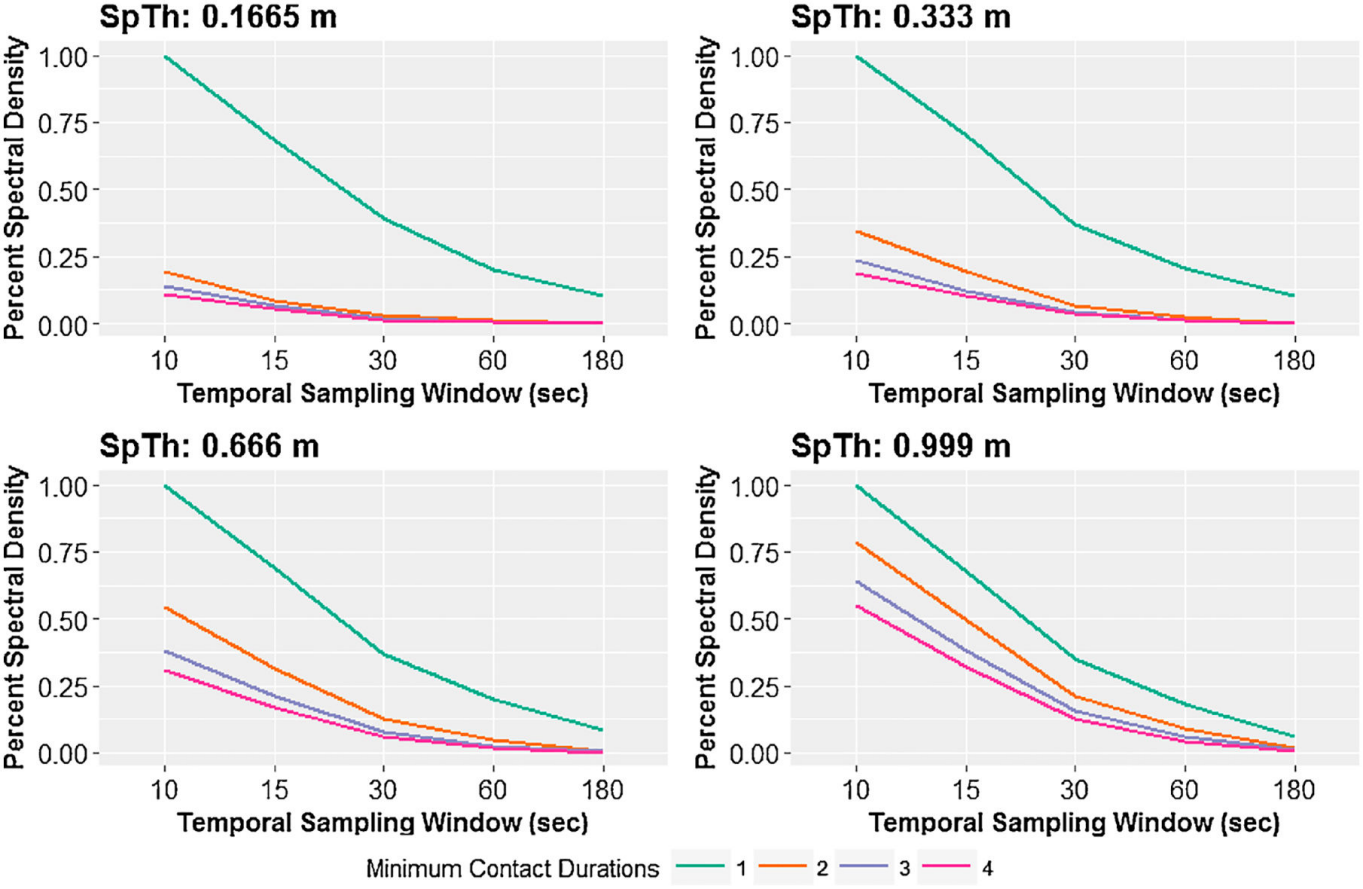
Percent spectral content per spatial threshold bin (0.1665 m, 0.333 m, 0.666 m, and 0.999 m) for data aggregated over hours. At each bin, percent spectral density is represented on the y-axis, temporal sampling windows are on the x-axis, and minimum contact durations are represented as different colored lines.

For a given SpTh value, the percent spectral density decayed monotonically with increases in both TSW and MCD, and had similar patterns of decay when aggregated at either the hourly (Fig. 2) or daily (S6) level. In general, the change in percent of spectral content was predictable between TSWs at a particular MCD, scaling with information content of the lowest TSW. Spectral content decay with increasing MCD was generally characterized by a large drop in percent spectral content from MCD = 1 to MCD = 2, followed by a more gradual loss of information content as MCD further increased. However, the quantity of decrease in spectral density with increases in MCD depended upon SpTh, with larger drops occurring as SpTh decreased.

**Fig. 3.**
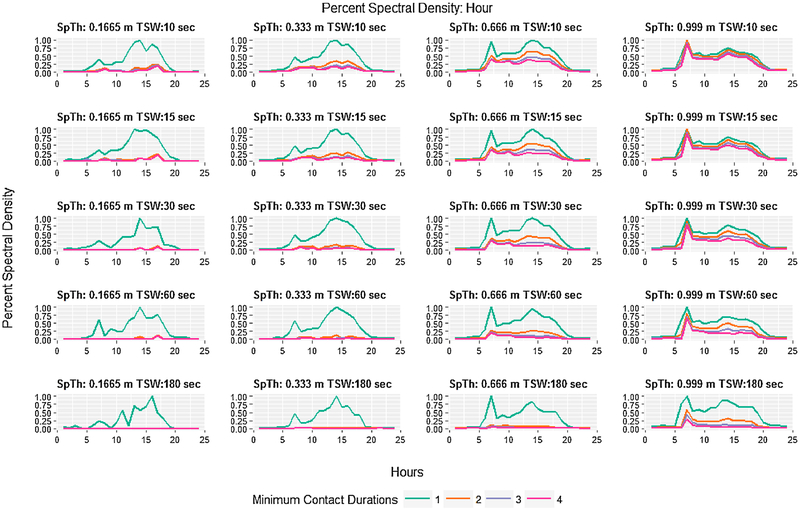
Average percent spectral content aggregated by hour and displayed over 24 h for all combinations of spatial threshold (ST), temporal sampling window (TSW), and minimum contact duration (MCD). The percent spectral content is indicated on the y-axis of each graph, and the hour is located on the x-axis. Parameter combinations for SpTh and TSW are shown above each graph, and MCD is indicated by colored lines.

**Fig. 4.**
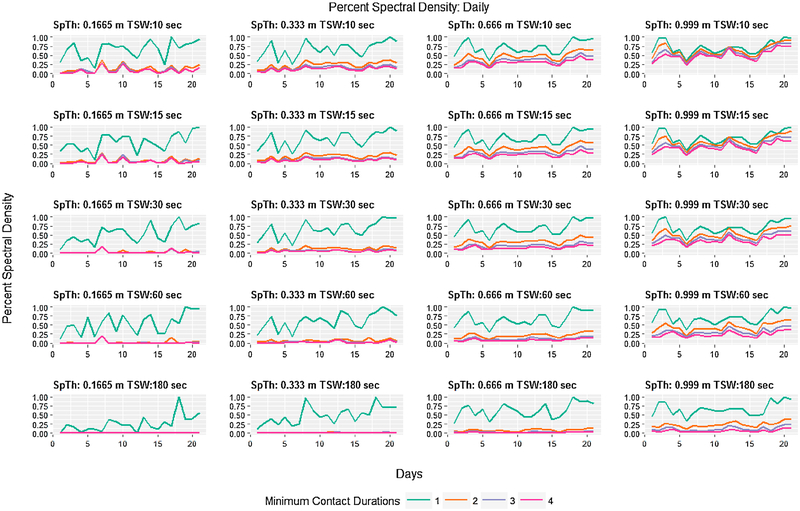
Average percent spectral content aggregated by day and displayed over 21 days for all combinations of spatial threshold (ST), temporal sampling window (TSW), and minimum contact duration (MCD). The percent spectral content is indicated on the y-axis of each graph, and the day is located on the x-axis. Parameter combinations for SpTh and TSW are shown above each graph, and MCD is indicated by colored lines.

Visualizing spectral density as a time series of hours or days revealed how daily and hourly patterns of contact information are preserved. First, spectral density and the effect of MCD on spectral density varied over hours in a manner that appeared to correspond to periods of low and high daily activity (Fig. 3). During periods of low activity (21:00 h–5:00 h, when cattle are behaviorally inactive, typically lying down), percent spectral density was similar across levels of MCD. During periods of higher activity (when cattle are eating, drinking, and socializing), the percent spectral density varied widely across hours, and large differences were apparent between when MCD = 1 and when MCD > 1 (Fig. 3). Two temporal patterns of note were a short spike around 6:00 h (feeding time), and then a long peak that begins around 7:30 h, reaches its zenith around 15:00 h and reaches its nadir shortly after 20:00 h. In general, as SpTh increased and TSW decreased, patterns of percent spectral density largely converged across all MCD. Conversely, as TWS increased and SpTh decreased datasets with MCD > 1 contained increasingly less information. In the most extreme case (SpTh = 0.1665 m, TSW = 180 s, MCD > 1), datasets contained almost no spectral density relative to a MCD = 1. At the daily scale a similar pattern of differences in spectral density with processing decisions was found (Fig. 4), and a cyclical pattern emerged in which a peak in percent spectral density occurred approximately every 5 days of the 21-day period.

A comparison to more typical contact metrics, average per-capita contact duration (number of contact durations per animal) per hour/day and average per-capita contacts (number of discrete contacts per animal) per hour/day (S7), showed that similar patterns were found with changes in processing decisions; that is, rates decreased as SpTh decreased, and as TSW and MCD increased. However, the decay of average per-capita contact duration (as a percent of the maximum for the SpTh level) occured more slowly than the decay of information via spectral density. For example, the percent spectral density for the combination (SpTh = 0.1665, TSW = 15, MCD = 2) aggregated at an hourly level was 8.5%, while the percent of maximum per-capita contact duration rate for the same combination was 25%. In contrast, the entropy measure was more robust to data loss than rates of contacts per hour/day. For example, at the same processing level, (SpTh = 0.1665, TSW = 15, MCD = 2), the percent of the maximum per capita contact rate was 7.9%. Thus, while the entropy-based metric was more sensitive to information loss than rates of per capita contact duration, it was less sensitive than rates of per capita contacts.

### 3.2. Transmission dynamics

**Fig. 5.**
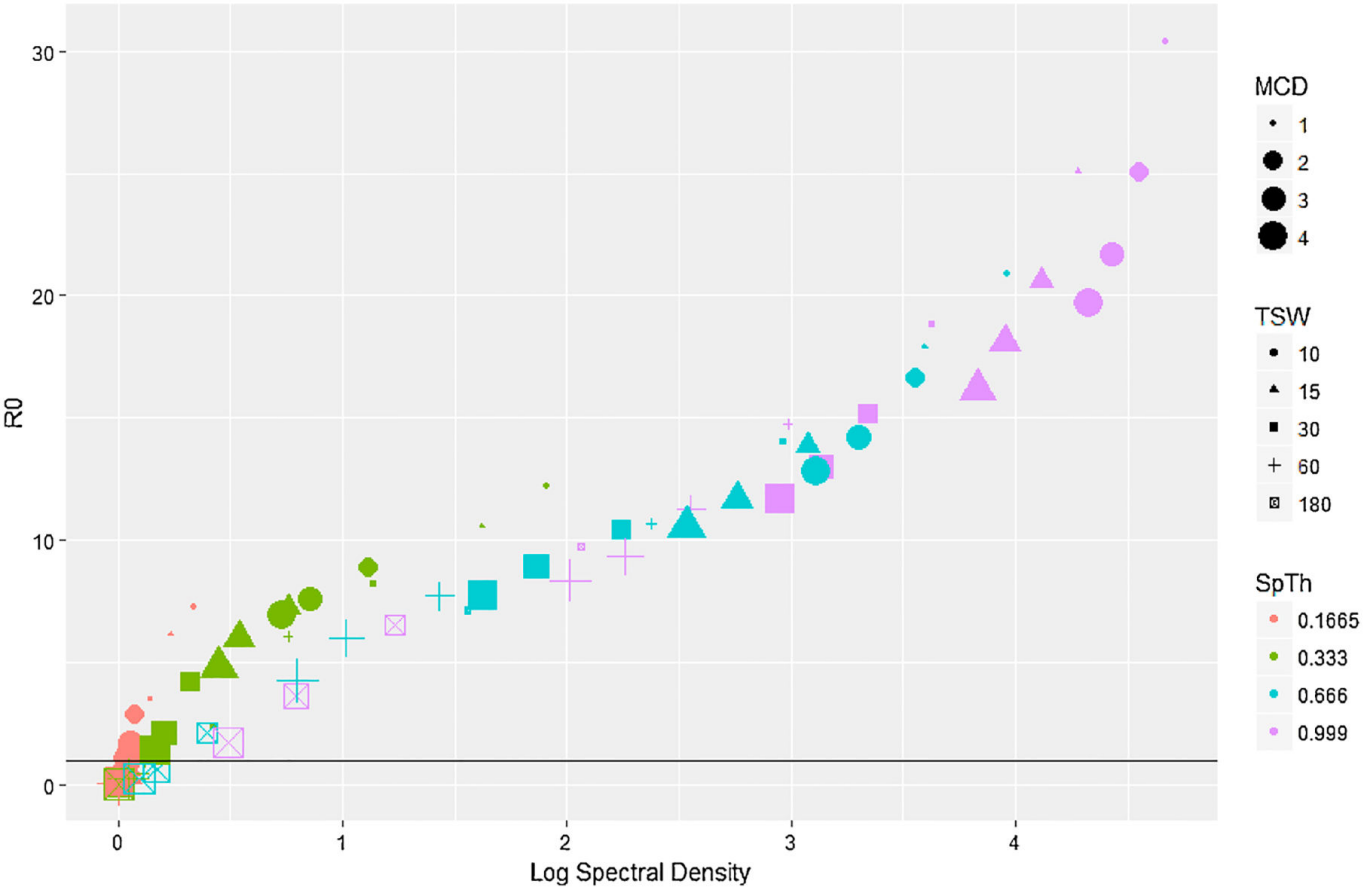
R_0_ as a factor of the average log spectral density of differently processed datasets aggregated at an hourly basis. SpTh value is indicated by color (orange = 0.1665 m, green = 0.333 m, blue = 0.666 m, purple = 0.999 m); TSW is indicated by shape (circle = 10 s; triangle = 15 s; square = 30 s; cross = 60 s; x-box = 180 s); MCD value is indicated by size.

**Fig. 6.**
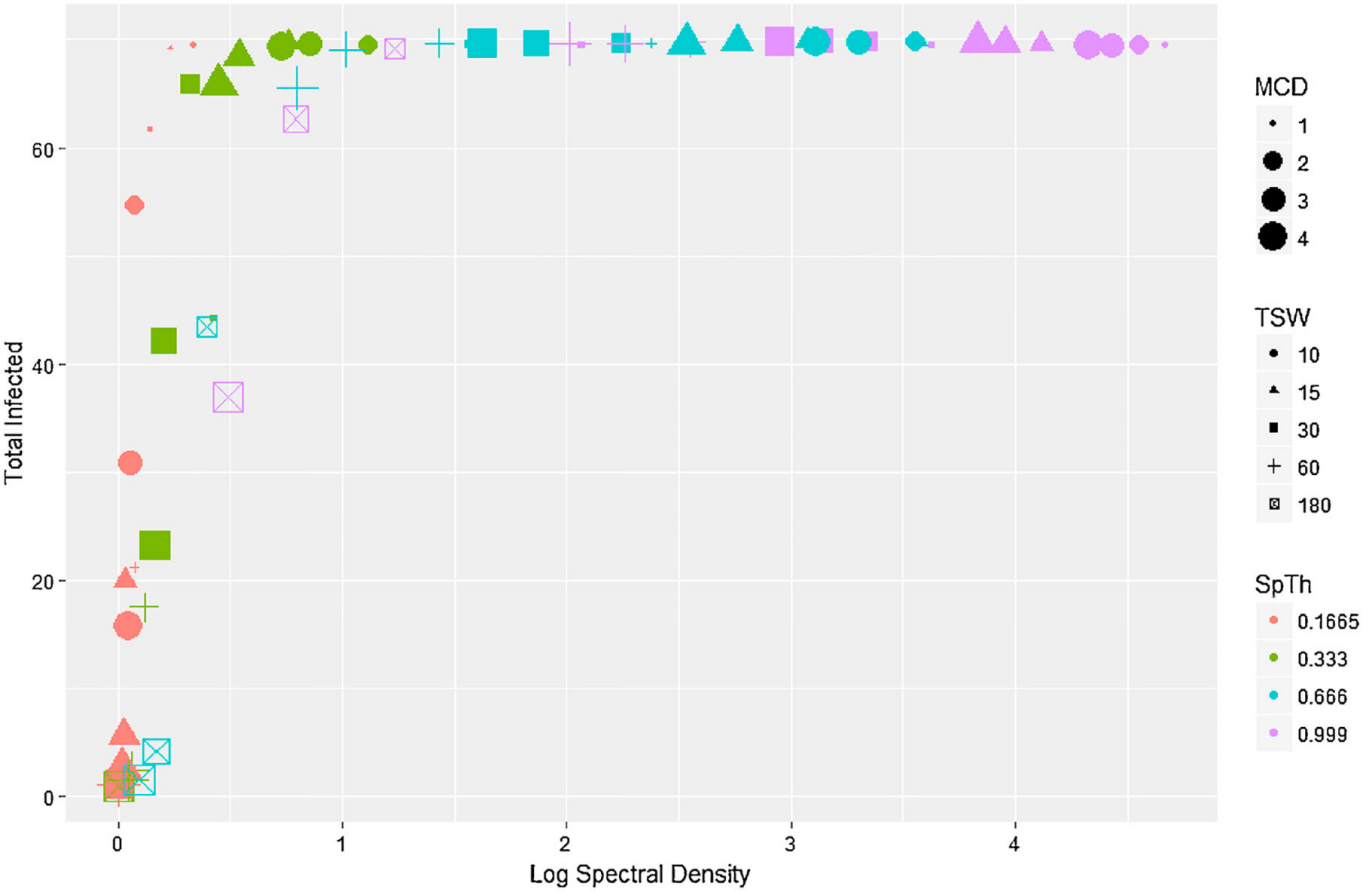
Total infected individuals as a factor of the average log spectral density of differently processed datasets aggregated at an hourly basis. SpTh value is indicated by color (orange = 0.1665 m, green = 0.333 m, blue = 0.666 m, purple = 0.999 m); TSW is indicated by shape (circle = 10 s; triangle = 15 s; square = 30 s; cross = 60 s; x-box = 180 s); MCD value is indicated by size.

**Fig. 7.**
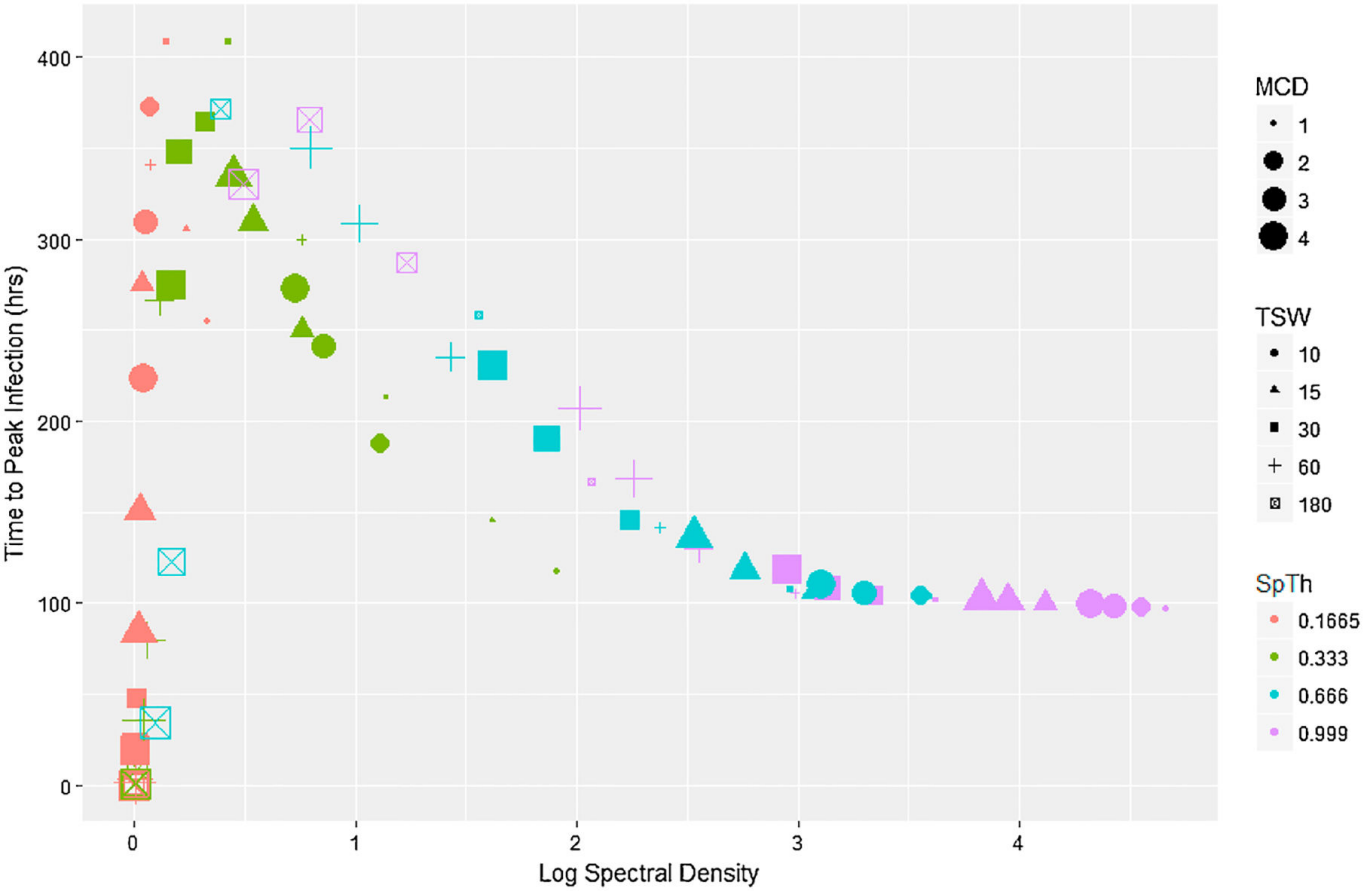
Peak infection time as a factor of the average log spectral density of differently processed datasets aggregated at an hourly basis. SpTh value is indicated by color (orange = 0.1665 m, green = 0.333 m, blue = 0.666 m, purple = 0.999 m); TSW is indicated by shape (circle = 10 s; triangle = 15 s; square = 30 s; cross = 60 s; x-box = 180 s); MCD value is indicated by size.

R_0_ was approximately linearly related to information content, increasing as (log) spectral density increased (Fig. 5), and particularly within a SpTh. A generalized linear model of R_0_ (S8) showed that this pattern was due to large main effects of, and relatively small interactive effects between processing decisions and R_0_. In contrast, the total number of infected individuals and time to peak infection were related in more complex ways with information content. The total number of infected individuals had a logistic relationship with information content, rapidly rising as log information spectral density increased, before leveling out at approximately complete infection once a particular threshold was reached (Fig. 6). A logistic regression of the probability of an epidemic conducted on total dichotomized total infected data (major epidemic (> 65 individuals infected) and minor epidemic (< 5 individuals infected)) demonstrated that this pattern was created via interactions between processing parameters. Namely, while increasing SpTh increased the probability of an epidemic, negative interactions with TSW and MCD caused it to rapidly fall (S8). Lastly, time to peak infection changed non-monotonically with information content, increasing with information content to a point, before decreasing (Fig. 7). A proportional hazards model(S8) showed that the first half of this pattern was produced by a positive interaction between TSW and MCD that reduced peak infection times as higher TSW and MCD values combined with low SpTh to reduce information content. This corresponded to simulations that failed to produce epidemics (Fig. 5) or had only minor outbreaks (Fig. 6). The second half of this pattern was due to the reduction of peak infection time with increasing SpTh (S8), and largely corresponded to major epidemics (Figs. 5 and 6).

Similar relationships between changes in information content and model metrics were found using daily aggregated data (S9.1) compared to hourly aggregated data, but quantitative differences in output parameters due to temporal aggregation existed. For R_0_, effect sizes of processing effects (calculated as: mean value_daily_-mean value_hourly_/st.dev._pooled_) (Nakagawa and Cuthill, 2007) ranged from −1.43 – 0.99 (i.e., higher R_0_ using hourly aggregated data with negative effect size), with R_0_ values higher using daily aggregated when information content was low, and higher with hourly aggregated data when information content was high (S9.2, Fig. 4). There was little difference in total cattle infected when information content was high (effect size = −0.14), and more cattle infected using daily-aggregated data when information content was low (effect size = 1.61) (S9.2, Fig. 5). Lastly, simulations using hourly aggregated data generally had lower (i.e., faster) times to peak infection (effect sizes −0.48–2.09), while most cases of higher peak infection time using hourly data were produced with datasets of lower information content (S9.2, Fig. 6). Statistical models showed that these patterns were due to variation in the magnitudes of main and interactive effects of temporal aggregation with processing parameters. For R_0_, interactive terms of similar magnitude were larger than the main effect for temporal aggregation, resulting in a pattern that depended on other processing parameters (S8). In contrast, main effects of temporal aggregation were larger than interactive effects for models of major epidemic occurrence and time to peak infection (S8), contributing to a range of effect sizes more tilted more towards daily or hourly aggregated data overall.

## 4. Discussion

### 4.1. Entropy and spectral density

Although processing decisions have been recognized previously as an important challenge when dealing with contact data (Eames et al., 2015; Haddadi et al., 2011), few researchers have approached the problem from the perspective of information loss (Haddadi et al., 2011). We extended the methodology proposed by Haddadi et al. (2011) by applying it in a factorial approach to consider how information content decays as a factor of multiple processing decisions. Entropy is a measure of the uncertainty of, and therefore the new information provided by, a value of a random variable (Stone, 2015). The entropy of adjacency matrices as calculated here reflects the uncertainty of the path of diffusion (of say, pathogens) over a graph (Gómez-Gardeñes and Latora, 2008; Sinatra et al., 2011), with greater entropy indicating easier diffusion (i.e., more paths). By converting time series of entropy values to a frequency basis and calculating an average spectral density per dataset, information captured over a time span was condensed into a single metric of information per period of temporal aggregation that allowed for a ready comparison between data processing methodologies (Haddadi et al., 2011). It showed that the information content of contact datasets drastically changed with differences in processing decisions (Fig. 2), but that temporal patterns tended to converge as entropy values increased (Figs. 3 and 4). As expected, a comparison to more straightforward contact metrics (rates per hour/day of average per-capita contact durations and discrete contacts) (S7) showed similar patterns of change with processing decisions. The entropy approach, however, explicitly accounts for network complexity, and is therefore less influenced by single pairwise contacts of long or short duration. Therefore, this technique provides a convenient method of assessing and comparing how processing decisions influence the information content of contact datasets and the potential for disease transmission over contact networks.

### 4.2. Relating processing decisions, spectral density, and disease transmission metrics

The disease model outputs considered here represented several measures of transmission dynamics, including the minimum threshold of contacts allowing major epidemics (R_0_), and the intensity (R_0_), rapidity (time to peak infection), and extent of epidemics (total cattle infected). Each was variably influenced by processing criteria, and whether or not a successful outbreak occurred. Successful epidemics occurred (e.g., R_0_ > 1 (Figs. 5 and 6)) over a wide range of processing parameter combinations, suggesting transmission threshold was remarkably robust to information loss. For example, R_0_ was only reduced to < 1 when > 92% of spectral density had been lost at the smallest SpTh (0.1665). At the largest SpTh (0.999 m), average R_0_ did not fall < 1 over the entire range of MCD and TSW considered, despite an information content of < 1% at the highest TSW and MCD combinations (Fig. 5). Additional simulations show that these conclusions generally hold across a wide range of dose rates (See S4.2).

When epidemics did occur, R_0_ increased in an approximately linear manner (Fig. 5) with log spectral density over a range from 1 – 42. Meanwhile, times to peak infection decreased in a logistic fashion from ≈ 429 h to a plateau of ≈ 100 h as information increased (Fig. 7). Thus processing decisions had a substantial effect on the rapidity and intensity of epidemics, with R_0_ more sensitive to changes in processing decisions than time to peak infection, particularly when information content is high. This is likely because model structure imposes a minimum time to when peak infection can occur (given an epidemic), regardless of information content. In contrast to R_0_ and time of peak infection, the final size of the outbreak was relatively insensitive to changes in processing parameters, with the majority of epidemics either major (> 65 individuals) or minor (< 5). Further, major outbreaks would likely result for epidemics of intermediate extent given a longer time. The implications of this are that particular attention should be paid to how contact data used in the model is processed if sensitive outputs like R_0_ are used to inform policy decisions.

Lastly, aggregating data at the hourly or daily level did result in differences in disease model outputs (S8, S9). Particularly when information content was higher (S9), faster epidemics (lower peak infection time) of higher intensity (higher R_0_) resulted with hourly data compared to daily data, likely due to the transmission function being evaluated more times with hourly (504) vs daily (21) datasets (Brouwer et al., 2017). However, when information content was lower, daily aggregation increased the number of contacts included in individual (i.e. daily) transmission trials, increasing R_0_ relative to hourly aggregated data (S9.2). Overall, however, the pattern of changes in output metrics with changing processing parameters was very similar between hourly and daily aggregated data (S9.1). Thus, our results are generally in concordance with Stehle et al. (2011) that reported aggregated contact data to be a good approximation to dynamic data. As suggested by Stehle et al. (2011), this may be due to the temporal scales of the epidemiological stages (days) being much longer than contact events (seconds to hours). In the case of quickly spreading pathogens, where the temporal scales of contact and transmission overlap, the level of temporal aggregation would be expected to have a larger influence on network topology and subsequent model dynamics.

One implication of our results is that since the quantity of information is influenced by processing decisions, epidemic patterns produced via modeling may be, in part, due to processing decisions alone. Therefore, networks created from different contact datasets, and subsequent model-based insights into disease dynamics derived from them may be less comparable if the datasets are processed in divergent ways. Previous research using dynamic contact data have employed a wide range of SpTh values as thresholds for contact. Thresholds for GPS-based data are often the largest (4–100 m) (Koen et al., 2017; Lavelle et al., 2014; Tosa et al., 2015; Vazquez-prokopec et al., 2013), and are due to technical limitations on ground-based readings. In the case of proximity sensors and real-time locations (such as the one in this study), thresholds typically have a much smaller range (0–2 m) (Barrat et al., 2014; Chen et al., 2014a; Drewe et al., 2012; Machens et al., 2013; Toth et al., 2015; Voirin et al., 2015). However, both contact structure (as indicated by spectral density) and disease dynamics in our study was shown to be very sensitive to changes in SpTh over a narrow range (0.1665–0.999 m), with its effect mediated by TSW and MCD. This suggests that diverse datasets should be re-processed to similar specifications if conclusions from their analyses are to be compared or datasets with different processing are used to inform a model.

We suggest that a general approach for the selection of processing criteria should start with the selection of an appropriate SpTh. Reasons for the selection of a SpTh are not always stated (Blyton et al., 2014), or are done so in the context of technical optimization (Duncan et al., 2012; Toth et al., 2015). However, SpTh had the largest independent influence on information content (Table 2) in our analyses, and featured prominently in statistical models of output metrics (Figs. 5–7, S8). In addition, it represents a hypothesis or knowledge about what is understood about the ecologies of the study organisms and about the dominant transmission pathway of the pathogen (Eames et al., 2015). In the case of proximity-sensory based approaches, this decision is often made prior to data collection, and may be as a function of the equipment used (Drewe et al., 2012; Obadia et al., 2015; Stehlé et al., 2011; Voirin et al., 2015). Therefore, careful consideration of the implications of varying SpTh on disease transmission, as well as trialing different settings to maximize performance (Drewe et al., 2012) may be necessary.

Next, data should be processed at the selected SpTh under varying TSW and MCD conditions. Although the transmission threshold of major epidemics (R_0_ > 1) was found to be fairly robust to processing decisions, there was little direct evidence as to what constituted appropriate versus excess levels of non-effective contacts within our datasets. A solution to reducing this uncertainty is through the analysis of epidemiologic data collected (ideally) contemporaneously with contact information (Manlove et al., 2018; Voirin et al., 2015). If there is a lack of comparative epidemiological data, three general strategies are apparent. The first is to assume that effective contacts can occur over a single temporal window of the shortest duration; that is, the dataset is used at its highest resolution. The second strategy assumes that some contacts are ineffective but there is high uncertainty as to reasonable level of processing stringency; thus, values for TSW and MCD are set to intermediate levels between their smallest and largest possible values. The third strategy assumes that most contacts are non-effective for disease transmission, and TSW and MCD are set to the largest values allowing an epidemic to occur (i.e., R_0_ > 1); that is, the dataset is used as its lowest effective resolution. Regardless of the strategy chosen to process a dataset, however, it appears essential that subsequent modeling efforts utilize the same processing methodology so that results are comparable.

### 4.3. Considerations

The conclusions presented here are subject to several limitations. First, our treatment of entropy was based on a TSW basis, wherein the entropy was calculated per time-ordered network snapshot at each TSW. This method did not explicitly consider how data-processing decisions influenced temporal correlations between particular topologies or sequences of contacts. However, temporal correlations can influence information flow, such as disease, over networks (Artime et al., 2017). An approach that takes this dependency into account uses “greedy” random walks to calculate entropy and quantify topological uncertainty (Saramäki and Holme, 2015). In greedy walks, walkers move between time steps based on the availability of contacts, choosing the next node at each time either randomly or a weighted basis. The probability of unique trajectories can be used to calculate a measure of entropy that represents the uncertainty of trajectories when starting from different nodes. When considered as a distribution, the effect of processing decisions on influencing temporal correlations can be assessed (Saramäki and Holme, 2015; Song et al., 2010). This aspect of data-processing influence on contact networks approach should be explored in future work.

Next, we developed a simulation model that used constant parameter values rather than sampling from distributions of parameter values to make interpretations of the influence of processing decisions clearer. However, the influence of additional stochasticity in model behavior may be important (Lloyd-Smith et al., 2005; Reynolds et al., 2015). In addition, exposure dose (CFUpcd) was also constant, with susceptibles accumulating CFUs through consecutive contacts with infectious individuals. Accounting for dose effect in another way may have influenced the rapidity and intensity of dynamics. For example, considering exposure dose by unit of discrete contact regardless of duration, for each consecutive TSW ≥ MCD, or by a variable rate depending on duration would likely have resulted in lower transmission.

The effect of the initially-infected individual was not explicitly considered in our main analysis, as the focus of the study was the effect of processing decisions on information content and model dynamics. Instead, we sought to reduce the effect of the initially-infected individual on transmission model dynamics by randomly selecting starting individuals for each simulation, and selecting an exposure rate (CFUpcd) that resulted in a moderate correlation between R_0_ and mean contact rate of the initially-infected individual during their infectious period (See S4.1). However, this correlation was based on the dataset with the highest information content, and further simulations (See S4.3) demonstrated that the effect of the initially infected individual on model dynamics is influenced by both infectious dose and how the dataset is processed prior to modeling efforts. In particular, the CFUpcd value for which the initially infected individual’s connectedness is maximally correlated with R_0_ (and therefore would be expected to have the greatest influence on model dynamics), changes in a non-linear way with information content, as determined by processing decisions (S4.3). In our simulations in which CFUpcd and starting individual were systematically varied (S4.2), the effects of processing decisions appear to be robust across a range of doses when R_0_ values were aggregated across all individuals within a factorial combination of processing decisions and dose. However, investigations into hypotheses on the influence of initially-infected individuals on disease dynamics should be cognizant of interactions between these factors.

Finally, there is uncertainty in how the relationship between information content, influenced by processing decisions, and disease dynamics would be affected by disease intervention strategies. Two factors that should be considered when assessing the plausibility of intervention-strategy implementation are behavioral modifications to the host that influence network structure such as sickness behavior (Eames et al., 2015), and the nature and efficacy of the intervention. Ideally, empirical contact data that captures both these aspects can be collected and modeled, leading to the creation of simulated contact data accounting for these issues (Pellis et al., 2015). In the absence of such quantitative information, understanding how data processing influences information content is helpful in gauging the uncertainty of intervention success. For example, the use of processing parameters that result in too little information may overestimate intervention success. Similarly, when interventions are less than 100% effective at reducing transmission, researchers must consider whether data processing leads to over- or under-estimated rates of simulated disease transmission compared to observed transmission. For example, a vaccine that provides only partial effectiveness, like recently developed *E.coli* O157 vaccines that reduce rates of shedding (Cull et al., 2012), may result in overly-optimistic simulation results if processing decisions over-reduce information content relative to reality. However, the relative insensitivity of the epidemic threshold (i.e., R_0_ > 1) to processing decisions shown here gives credence to the effectiveness of interventions that prevent (i.e., reduce transmission so that R_0_ < 1) otherwise successful simulated epidemics.

### 4.4. Conclusions

In this study, we found that upstream processing decisions influence the information content of contact datasets, resulting in significant implications to the dynamics of disease models. Using entropy as a concise descriptor of information content, we found that R_0_ and time to peak of infections were relatively sensitive to processing decisions, while transmission threshold (R_0_ > 1) and final outbreak size were less sensitive. In addition, model dynamics may be more sensitive to processing decisions if the temporal scales of contact formation and transmission are more likely to overlap. Overall, our findings emphasize that the selection of processing criteria can impose a considerable influence on contact structure and resulting transmission model dynamics, and thus should be selected in a cognizant, and non-trivial manner.

## Supplementary Material

1

2

3

4

5

6

7

8

9

## Figures and Tables

**Table 1:** Implications and visual explanations of processing criteria for dynamic contact data in disease transmission models.

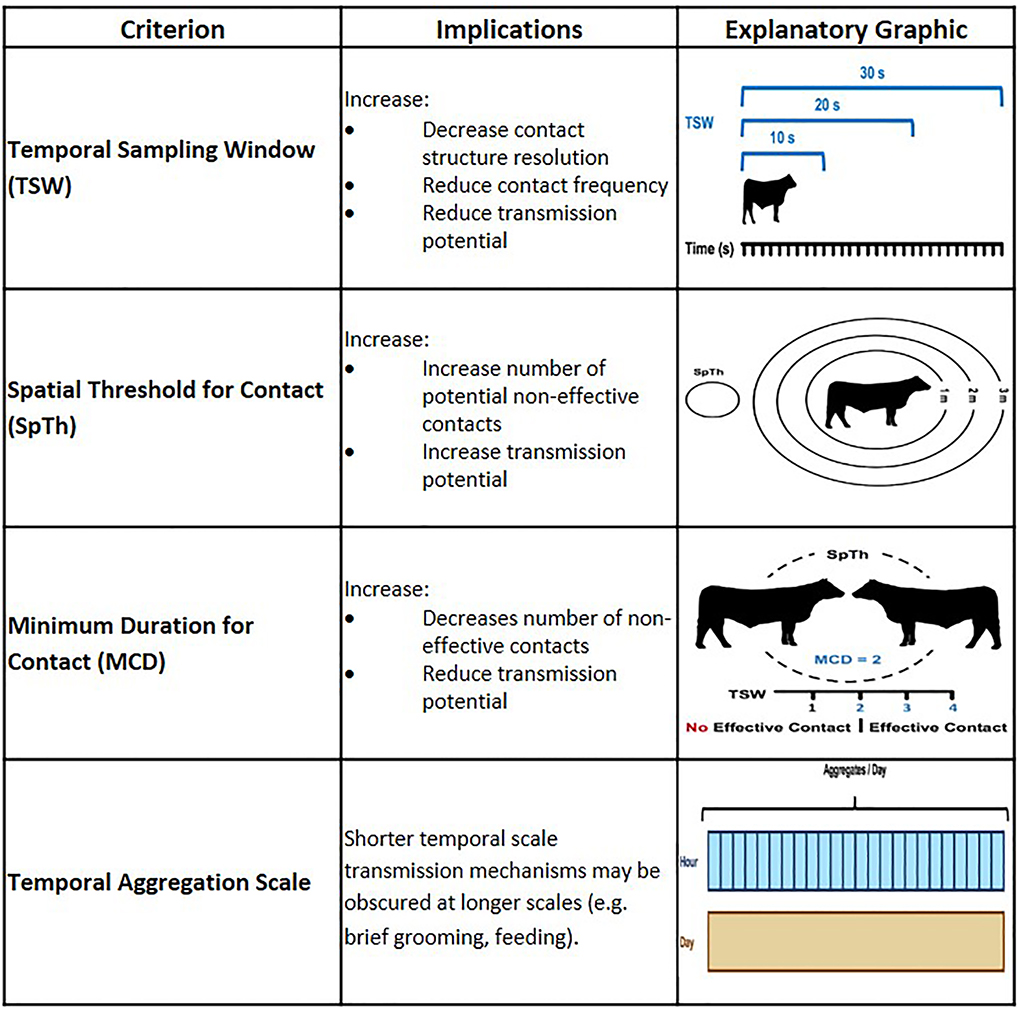

**Table 2:** Partial Ranked Correlation Coefficient (PRCC) analysis of Spatial Threshold (SpTh), Temporal Sampling Window (TSW), and the Minimum Contact Duration (MCD) on average spectral density aggregated over both hours and days.

Temporal Scale	Parameter	PRCC	95 % CI	
Hour	SpTh	0.721	0.716	0.726
	TSW	−0.428	−0.437	−0.421
	MCD	−0.320	−0.329	−0.312
Day	SpTh	0.959	0.956	0.962
	TSW	−0.832	−0.847	−0.820
	MCD	−0.657	−0.679	−0.634

## Abbreviations:

SpTh: Spatial Threshold of Contact
TSW: Temporal Sampling Window
MCD: Minimum Contact Duration
CFU: colony-forming units
CFUpcd: colony-forming units per contact duration
