# Supplementary material for: Transmission on empirical dynamic contact networks is influenced by data processing decisions
Source: Epidemics. Author manuscript; Available in PMC 2019 Jul 8. (PMC6613374; doi:10.1016/j.epidem.2018.08.003)
Supplement: 1 [file NIHMS1526165-supplement-1.zip › S1_Dawson et al.2018_ProcessingDetails.docx]

S1.Preprocessing and Processing Procedures

S1.1 Pre-processing

Prior to processing into temporal sampling periods, raw location data was pre-processed in two main ways for quality control purposes. First, radio-signal based movement data is susceptible to interference, resulting in locations appearing to move rapidly, or bounce, from one location to another. In this dataset, this occurred for all individuals on an occasional basis, and more frequently for particular individuals, potentially due to problems with individual transmitters. To account for overall bounce issues, all records for individual cattle were filtered to limit sequential movements to those requiring an average velocity of no greater than 10 meters per second by cattle, considered a reasonable top speed for feed lot cattle. Second, some individuals had a high degree of bounce issues, even when a 10 m/sec filter was applied. These individuals were identified by quantifying distances between all sequential movements of all individuals, binning movements into 5 m categories (5,10,15,20,25,>30), and calculating the percentage of movements in each bin. From this approach, it was found that while sequential movements by most individuals on most days were overwhelmingly (≈90%) small (<5 m), some individuals exhibited a large percentage of long distance movements (10-25%, >25 m). Investigating the underlying locational data of these high movement individuals demonstrated a high degree of “bouncing” between particular locations, indicative of reception/transmission issues. To account for these individuals, an ad hoc decision was made to exclude data from individual cattle on particular days if they had a high proportion of bounce locations, defined here as having > 1% of sequential movements ≥ 25 m. This resulted in the removal of 25 daily cattle records, or 1.7% of the total daily cattle records (70 cattle * 21 days).

S1.2 Processing Procedures

To begin, movement records of sequential locations of individuals (and therefore different numbers of records per individual) were made more easily comparable by smoothing them into interpolated locations per second. This interpolation was done by backwards applying locations (that is, all of the seconds between location_t_ and location_t+1_ were assigned location_t+1_), with the reasoning that because locations were transmitted upon movement, the animal could be assumed to be at the last location it was recorded at until it moved. Second, temporally smoothed data were sorted into a series of sampling windows, including 10 seconds, 15 seconds, 30 seconds, 60 seconds, and 180 seconds. The lower bound of 10 seconds was due to data being collected at a maximum frequency of 5-10 seconds. Temporal sampling was accomplished by averaging x and y locations within each sequential temporal interval (e.g., for the 10 sec sampling window, locations within each of 8640 intervals per day were computed by averaging locations in each sequential 10 second window). Next, contacts between individuals were computed by first calculating the distances between all individuals at each temporal sampling window (TSW) using Euclidean methods (Chen et al., 2013). Then, a contact was determined as occurring within a TSW if the distance was ≤ to a spatial threshold (SpTh). Spatial thresholds considered ranged from 0.1665 m, 0.333 m, 0.666 m, and 0.999 m, and thus included both conservative (≈0.3 head length), moderate, and liberal ( ≈ 0.5 body length) criteria for contact as it relates to directly-transmitted disease risk. Third, contact records were filtered based on whether they were of a minimum consecutive duration length. Minimum duration criteria of 1,2,3, and 4 consecutive temporal sampling windows were considered, and thus included criteria of no truncation (i.e., 1 interval) to strong truncation (at least 4 consecutive intervals). Lastly, contact information was used to create adjacency matrices between all individuals at each time step for the dataset created for each parameter combination (n=80). For the transmission models, contact matrices were assembled from contact data aggregated at each hour and each day.

References

Chen, S., Sanderson, M.W., White, B.J., Amrine, D.E., Lanzas, C., 2013. Temporal-spatial heterogeneity in animal-environment contact: Implications for the exposure and transmission of pathogens. Sci. Rep. 3, 1–6. doi:10.1038/srep03112
