# Supplementary material for: Transmission on empirical dynamic contact networks is influenced by data processing decisions
Source: Epidemics. Author manuscript; Available in PMC 2019 Jul 8. (PMC6613374; doi:10.1016/j.epidem.2018.08.003)
Supplement: 4 [file NIHMS1526165-supplement-4.zip › S4_Dawson et al.2018_Determining CFUpcd_dose effects.docx]

S4.1: Parameterization of CFU per contact duration parameter

Preliminary work had demonstrated that individual cattle varied in their contact rates with other cattle. Since simulations started with a single infectious individual, the selection of the initially infected individual could potentially have an important impact on subsequent infection dynamics. However, the intent of the study was to determine how processing decisions influenced overall contact structure and transmission dynamics. Thus, it was desirable to select a CFUpcd that would reduce influence of the initially infected individual on disease transmission dynamics.

To account for this, we ran a series of simulations in which the starting individual was systematically varied over the entire set (n=70), and over a wide range of CFUpcd values (1,2,4,6,8,10,100,500,1000,5000,10000) for 10 replicates a piece; a total of 7700 simulations. For these simulations, we used the parameter combination in which SpTh = 0.999 m, TSW = 10 m, and MCD =1, identified as having the largest amount of spectral density. For each CFUcd, Pearson correlations were calculated and plotted between the average R_0_ and the total average number of contacts over the infectious period for the starting individual (10 days). To facilitate interpretation over the wide range of values considered, CFUpcd values were converted to a log scale for plotting.

Correlations between contact rates and R0 values decreased rapidly as CFUpcd increased from 1-10 (untransformed), and then decreased more slowly as CFUpcd ranged from ranged from 10-10000 (untransformed) (Fig. S4.1). This indicated that connectivity of the initially infected individual was highly influential to the dynamics of resulting model simulations when CFUpcd was low, but decreased as CFUpcd increased. Because the influence of the initially infected individual was relatively stabilize between CFUpcd 10-500, we selected 100 CFU per duration (4.61 on the log scale) to use in factorial simulations.

This value falls significantly lower than that often administered as an infectious dose during experimental trials in cattle (300-10^10) for pathogens such as Escherichia coli 0157 (Besser et al., 2001; Cray and Moon, 1995; Kulow et al., 2012; Sheng et al., 2016). However, environmental exposures are likely to be highly variable and much less than experimental doses, which are purposely high to ensure colonization. In addition, 100 CFUpcd was clearly more than sufficient to allow epidemics to occur, as indicated by high mean R_0_ values at this level (Fig. S1.3).


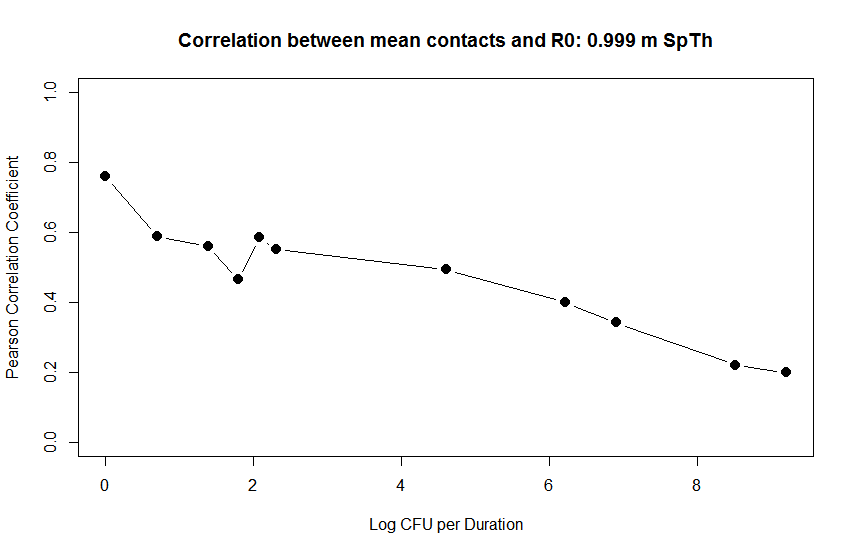


Figure S4.1. Correlation between mean contacts per day of the initially infected individual and R_0_ of simulated epidemics as a function of log CFUpcd.

S4.2. Characterizing the effect of CFUpcd on disease dynamics

To assess how CFUpcd interacts with processing decisions to influence the information content of contact networks and subsequent outputs of disease transmission models, we used the methodology described above but expanded it to include other factorial combinations of processing decisions. Specifically, we included factorial combinations at each SpTh producing the highest and lowest information content, namely TSW=10 sec and 180 sec, and MCD = 1 and 4 (16 factorial combinations). We included the same range of CFUpcd as above, and conducted 10 simulation replicates in which each individual was selected as the initially infected individual, a total of 123200 simulations. Next, R0 was extracted from each simulation, and averaged over all simulations within a given factorial combination of processing decision and CFUpcd. Lastly, these R0 values were plotted against the Log Spectral Density of the corresponding factorial combination of processing factors using the information content of the first 10 days (the infectious period ) of the simulation (A), and from the entire temporal span (21 days) of the dataset. These figures show that although dose significantly interacts with information content to influence disease model output, conditions preventing epidemics from occurring included a relatively narrow range of low doses(<10 CFUpcd) and information conditions. In addition, Figures S4.2.1A & B are very similar, showing that the information content within the first infectious period served as a good indicator of final disease dynamics. This likely occurred here because the simulation period was relatively short (21 days) compared to the timescale of epidemiologic parameters (recovery period = 10 days, latency period = 1 day). Further, even though we noted a periodic pattern in spectral density over the days of the dataset (Fig. 5), the pattern was similar between the first and second halves of the simulation period. For diseases that progress over a more rapid time scale, or when contact patterns display distinct temporal patterns, similar findings may not be expected to hold.


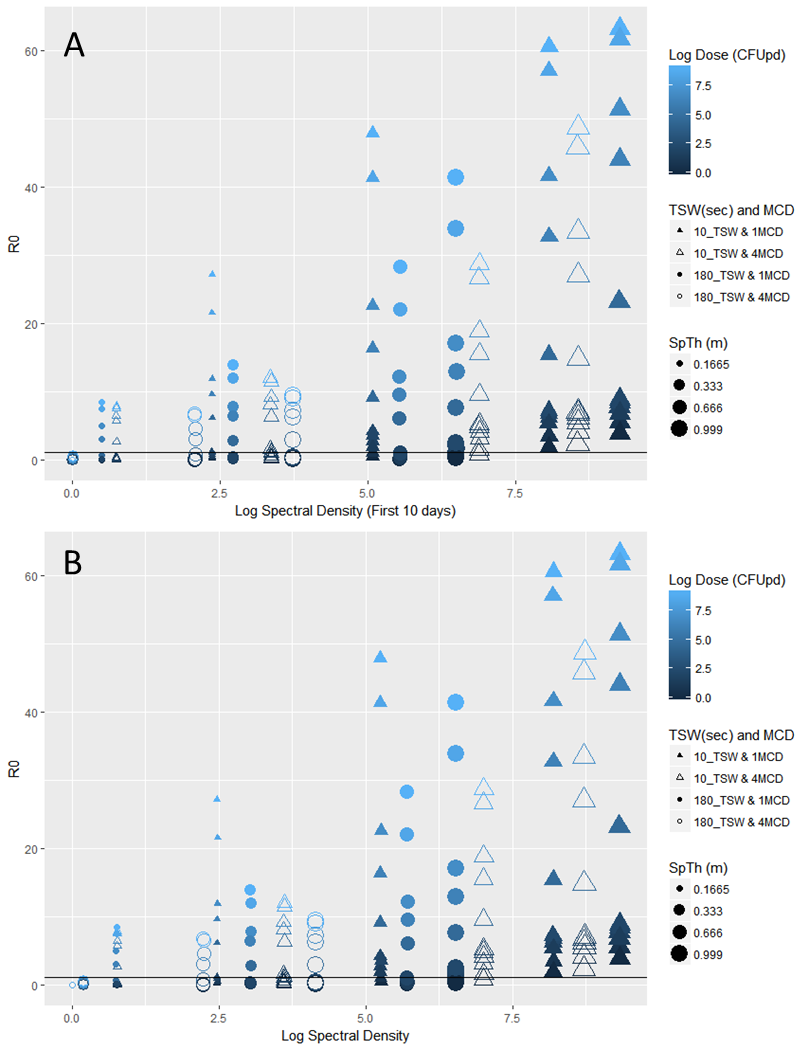


Figure S4.2. R0 as a factor average log Spectral Density over the first 10 days (A) and the entire temporal period (B) of the dataset. Dose (ranging from 1-10000) is represented from dark to light blue, processing level within a SpTh is represented by shape (triangle: 10 TSW; circle: 180 TSW; filled: 1 MCD; open: 4 MCD), and SpTh is represented by size, ranging from 0.1665 m (smallest) to 0.999 m (largest). The level of R0 indicative of a successful epidemic (R0>1) is shown as a solid line. Most dose and processing combinations result in R0>1. Dose clearly influences dynamics, but only low dose and/or low spectral density combinations result in no epidemics.

S4.3: Characterizing the effect of processing parameters on the influence of initially effected individual

Utilizing the simulations generated is S4.2, we expanded upon the methodology described in S4.1 to quantify the relationship between the connectivity of the initially infected individual, dose, and disease dynamics. At each SpTh, Pearson Correlation Coefficients were calculated and plotted over log CFUpcd (Figure S4.3A-D). The figures demonstrate that the relationship between the CFUpcd, and the correlation between R0 and connectivity is variable depending on how the dataset was processed. In particular, there appears to be a CFUpcd at a particular SpTh at which this association is highest, which tends to increase as information content decreases; namely, as TSW and MCD become larger.


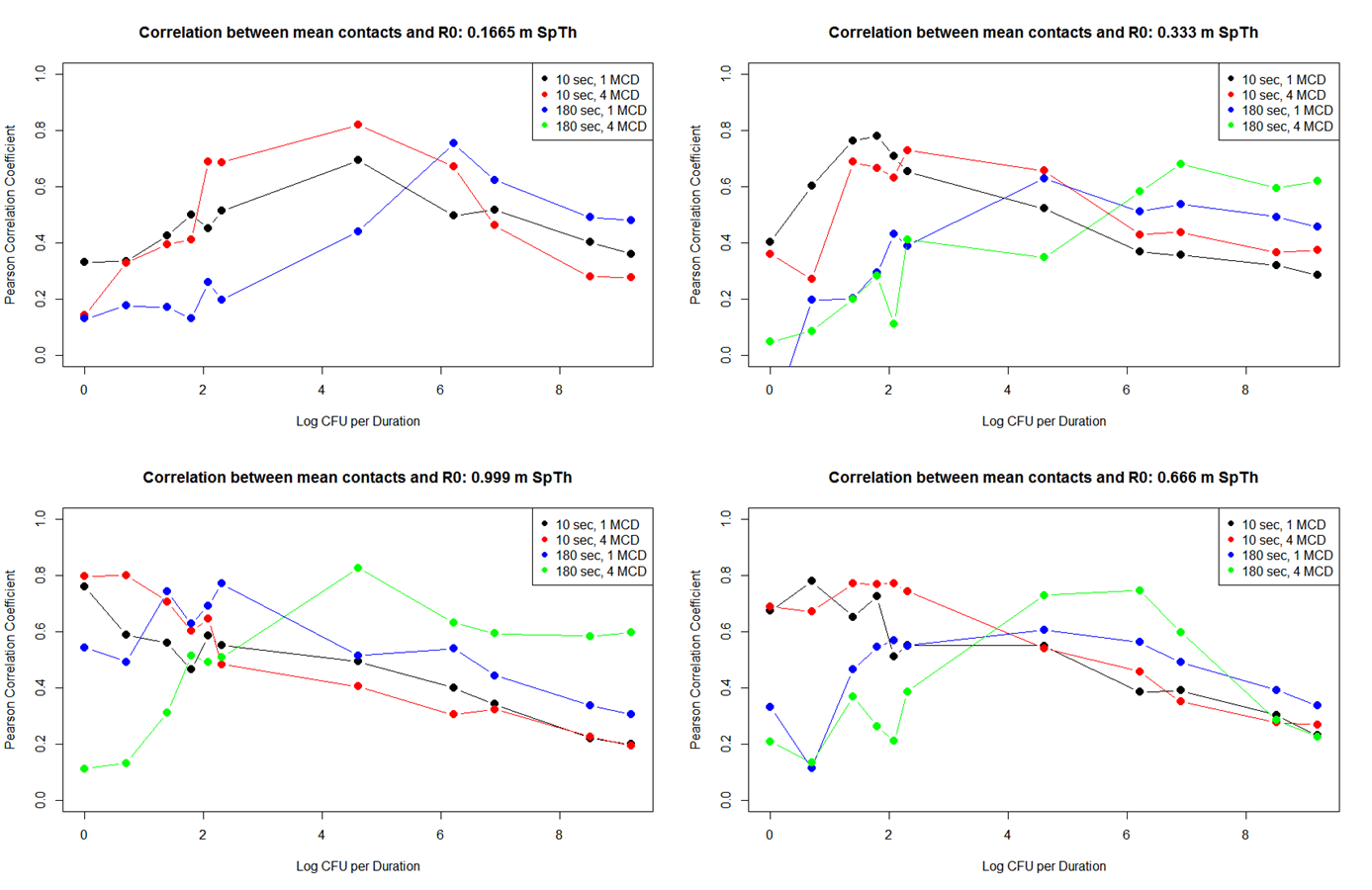


Figure S4.3.1. Correlations between mean contacts per day of the initially infected individual and R_0_ of simulated epidemics as a function of log CFUpcd at each SpTh (top row: 0.1665 m, 0.333 m; bottom row: 0.666 m; 0.999 m). At each SpTh, colors indicate factorial processing combination; black = 10 sec TSW, 1 MCD; red = 10 sec TSW, 4 MCD; blue = 180 sec TSW; 1 MCD; green = 180 sec TSW; 4 MCD).

**References**

Besser, T.E., Richards, B.L., Rice, D.H., Hancock, D.D., 2001. Escherichia coli O157:H7 infection of calves: infectious dose and direct contact transmission. Epidemiol Infect 127, 555–560. doi:10.1017/S095026880100615X

Cray, W.C., Moon, H.W., 1995. Experimental infection of calves and adult cattle with Escherichia coli O157:H7. Appl. Environ. Microbiol. 61, 1586–1590.

Kulow, M.J., Gonzales, T.K., Pertzborn, K.M., Dahm, J., Miller, B.A., Park, D., Gautam, R., Kaspar, C.W., Ivanek, R., Döpfer, D., 2012. Differences in colonization and shedding patterns after oral challenge of cattle with three Escherichia coli O157:H7 strains. Appl. Environ. Microbiol. 78, 8045–8055. doi:10.1128/AEM.02363-12

Sheng, H., Shringi, S., Baker, K.N.K., Minnich, S.A., Hovde, C.J., Besser, T.E., 2016. Standardized Escherichia coli O157 : H7 Exposure Studies in Cattle Provide Evidence that Bovine Factors Do Not Drive Increased Summertime Colonization 82, 964–971. doi:10.1128/AEM.02839-15.Editor
