# Supplementary material for: Transmission on empirical dynamic contact networks is influenced by data processing decisions
Source: Epidemics. Author manuscript; Available in PMC 2019 Jul 8. (PMC6613374; doi:10.1016/j.epidem.2018.08.003)
Supplement: 6 [file NIHMS1526165-supplement-6.zip › S6_Dawson et al.2018_PSD_Daily_Aggregation.docx]

Supporting Information 6: Percent of the maximum spectral density of datasets aggregated at a daily level


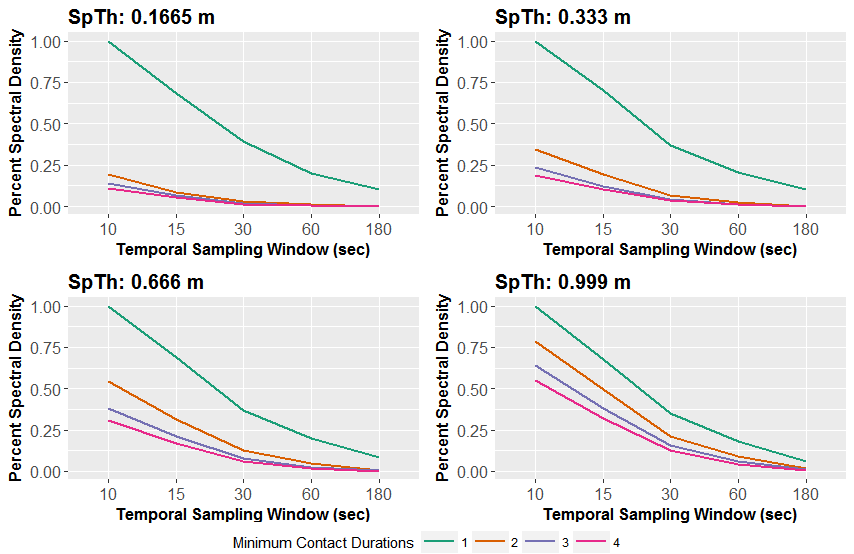


Percent spectral content per spatial threshold bin (0.1665 m, 0.333 m, 0.666 m, and 0.999 m) for data aggregated over days. At each bin, percent spectral density is represented on the y-axis, temporal sampling windows are on the x-axis, and minimum contact durations are represented as different colored lines.
