# Supplementary material for: Transmission on empirical dynamic contact networks is influenced by data processing decisions
Source: Epidemics. Author manuscript; Available in PMC 2019 Jul 8. (PMC6613374; doi:10.1016/j.epidem.2018.08.003)
Supplement: 7 [file NIHMS1526165-supplement-7.zip › S7.1_Dawson et al.2018_Contact Metrics_Figures.docx]

Supporting Information 7.1: Percent of per capita contact durations


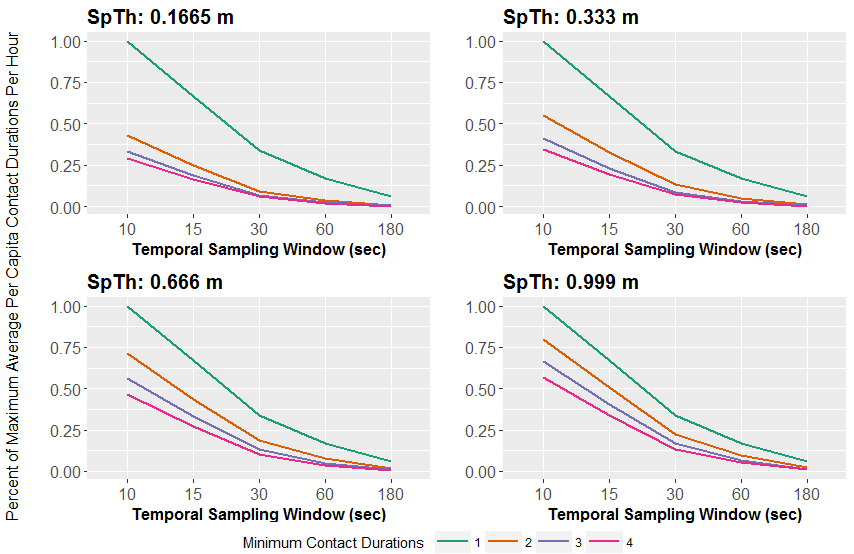


Figure 1: Percent of maximum of average per capita contact durations (i.e. average number of contact durations per animal) per spatial threshold bin (0.1665 m, 0.333 m, 0.666 m, and 0.999 m) for data aggregated over hours. At each bin, percent maximum average per capita contact durations is represented on the y-axis, temporal sampling windows are on the x-axis, and minimum contact durations are represented as different colored lines.


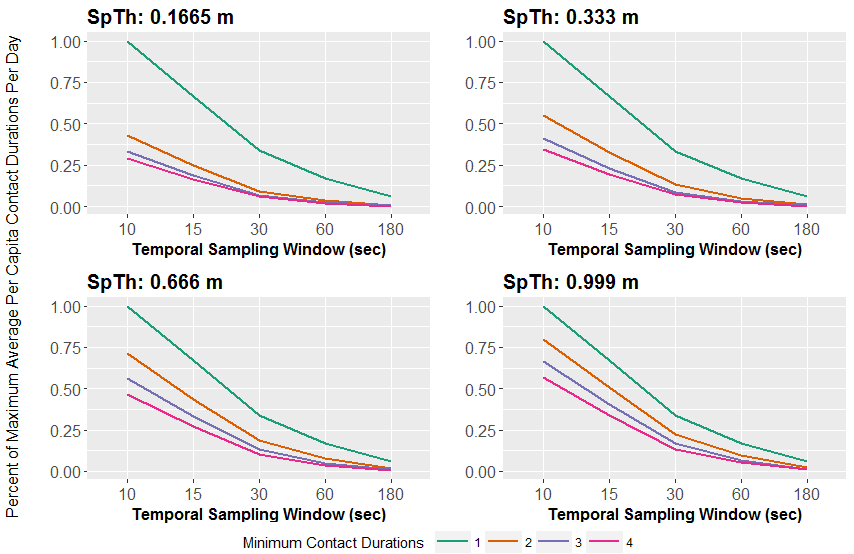


Figure 2: Percent of maximum of average per capita contact durations (i.e. average number of contact durations per animal) per spatial threshold bin (0.1665 m, 0.333 m, 0.666 m, and 0.999 m) for data aggregated over days. At each bin, percent maximum average per capita contact durations is represented on the y-axis, temporal sampling windows are on the x-axis, and minimum contact durations are represented as different colored lines.


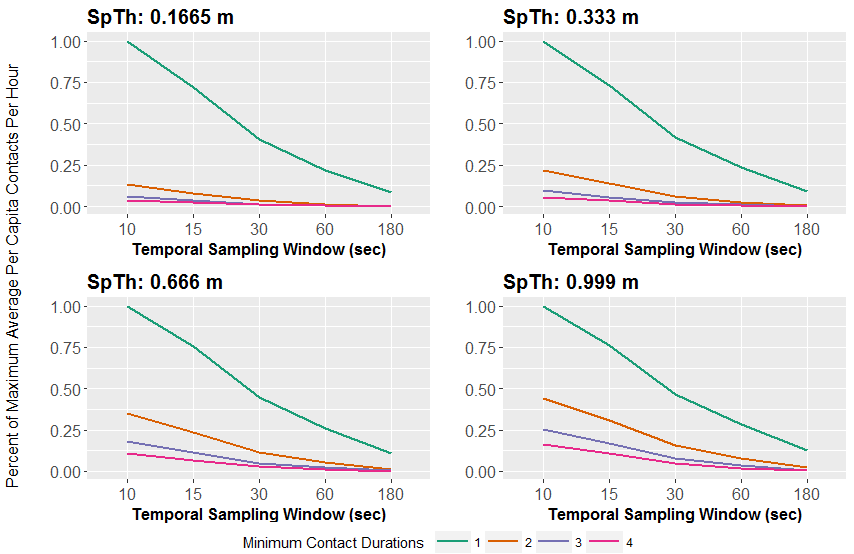


Fig 3: Percent of maximum of average per capita contacts (i.e. average number of discrete contacts per animal) per spatial threshold bin (0.1665 m, 0.333 m, 0.666 m, and 0.999 m) for data aggregated over days. At each bin, percent maximum average per capita contact durations is represented on the y-axis, temporal sampling windows are on the x-axis, and minimum contact durations are represented as different colored lines.


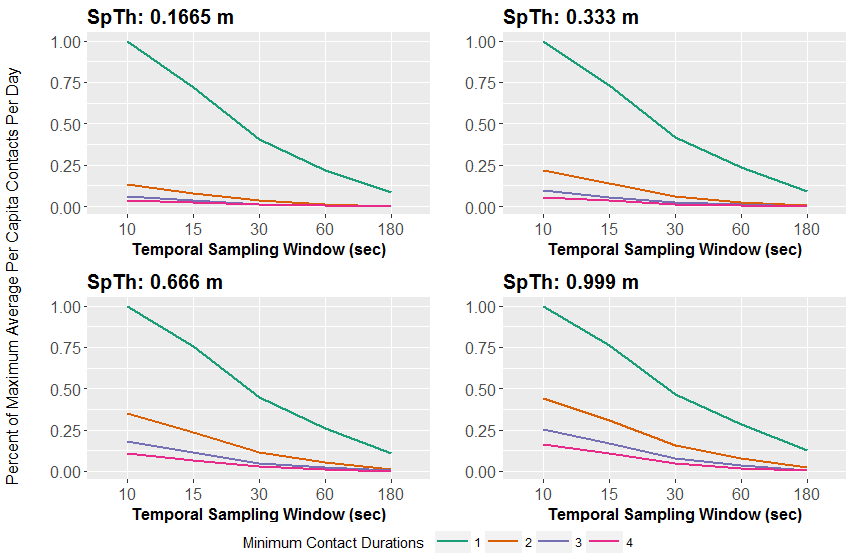


Fig 4: Percent of maximum of average per capita contacts (i.e. average number of discrete contacts per animal) per spatial threshold bin (0.1665 m, 0.333 m, 0.666 m, and 0.999 m) for data aggregated over days. At each bin, percent maximum average per capita contact durations is represented on the y-axis, temporal sampling windows are on the x-axis, and minimum contact durations are represented as different colored lines.
