# Supplementary material for: Transmission on empirical dynamic contact networks is influenced by data processing decisions
Source: Epidemics. Author manuscript; Available in PMC 2019 Jul 8. (PMC6613374; doi:10.1016/j.epidem.2018.08.003)
Supplement: 8 [file NIHMS1526165-supplement-8.zip › S8_Dawson et al.2018_Statistical Model Analysis.docx]

Supporting Information 8: Statistical models of each output as a factor of processing criteria.

To investigate the role of processing parameters in influencing disease model outputs, each output (R_0_, total cattle infected, time to peak infection) was modeled using a different statistical approach. Each model consisted of all main (4), and 2-way interactive effects (6). Parameter values for processing decisions were centered and scaled to 1 standard deviation prior to analysis to facilitate model interpretation. R_0_ was modeled using a general linear mixed model with a random intercept for the initially infected individual in the simulation (S8, Table 1) (N=160,000). The distribution of total cattle infected was strongly bi-modally distributed toward either 1 or 70 (i.e., no secondary infections occurred, or all cattle became infected). To model this pattern, data were split into two groups (representing approximately 89% of the total dataset (N=142,029) consisting of simulations resulting in minor epidemics (secondary infections ≤ 5) and simulations with major epidemics (secondary infections ≥65). These two groups were then included in a logistic regression model of the probability of an epidemic (defined here as all or nearly all individuals becoming infected) occurring (S8: Table 2). Lastly, the time to peak infection (hrs.) was modeled using a Cox proportional hazards model stratified by the initially infected individual (N=160,000) (S8: Table 3). All analysis was done using program R (R Core Team, 2017). The GLMM was fit using package lme4 (Bates et al., 2015), the GLM was fit using the stats package (R Core Team, 2017), and the Cox’s proportional hazards model was fit using the survival package (Therneau, 2015).

| **Fixed Effects** |  |  |  | 95% CI |  |  |
| --- | --- | --- | --- | --- | --- | --- |
| Variable | Estimate | St. Error | t | *LB* | *UB* |  |
| Intercept | 1.631 | 0.016 | 104.600 | 1.600 | 1.661 |  |
| TSW | -0.473 | 0.002 | -270.500 | -0.477 | -0.470 |  |
| SpTh | 0.616 | 0.002 | 351.800 | 0.612 | 0.619 |  |
| MCD | -0.258 | 0.002 | -147.300 | -0.261 | -0.254 |  |
| Hourly Aggregation | 0.007 | 0.002 | 2.700 | 0.002 | 0.012 |  |
| TSW:SpTh | -0.022 | 0.001 | -17.900 | -0.025 | -0.020 |  |
| TSW:MCD | -0.051 | 0.001 | -40.900 | -0.053 | -0.048 |  |
| SpTh:MCD | 0.051 | 0.001 | 41.400 | 0.049 | 0.054 |  |
| SpTh:Hourly Aggregation | 0.152 | 0.002 | 61.400 | 0.147 | 0.157 |  |
| TSW:Hourly Aggregation | -0.064 | 0.002 | -26.000 | -0.069 | -0.060 |  |
| MCD:Hourly Aggregation | -0.087 | 0.002 | -35.200 | -0.092 | -0.082 |  |
| **Random Effects** |  |  |  |  |  |  |
| Groups | Std.Dev. |  |  |  |  |  |
| Initially Infected Individual | 0.1278 |  |  |  |  |  |
| Residual | 0.4949 |  |  |  |  |  |

Table1. Results of a GLMM of R_0_ (log transformed) in disease transmission models simulated using contact data processed in factorial combinations of temporal sampling window (TSW), minimum contact duration (MCD), spatial threshold (SpTh), and temporal aggregation (Hourly and Daily). “:” indicates interaction between two variables.

|  |  |  |  | 95% CI | |
| --- | --- | --- | --- | --- | --- |
| Variable | Estimate | St. Error | z | *LB* | *UB* |
| Intercept | 6.196 | 0.111 | 55.820 | 5.978 | 6.413 |
| TSW | -11.353 | 0.158 | -71.958 | -11.662 | -11.044 |
| SpTh | 14.986 | 0.202 | 74.054 | 14.590 | 15.383 |
| MCD | -2.564 | 0.064 | -40.349 | -2.689 | -2.440 |
| Hourly Aggregation | -1.730 | 0.103 | -16.823 | -1.932 | -1.529 |
| TSW:SpTh | 4.489 | 0.100 | 44.684 | 4.292 | 4.686 |
| TSW:MCD | -8.811 | 0.148 | -59.648 | -9.100 | -8.521 |
| SpTh:MCD | 5.061 | 0.102 | 49.851 | 4.862 | 5.260 |
| SpTh:Hourly Aggregation | 0.414 | 0.151 | 2.748 | 0.119 | 0.710 |
| TSW:Hourly Aggregation | -0.184 | 0.099 | -1.859 | -0.377 | 0.010 |
| MCD:Hourly Aggregation | -1.270 | 0.069 | -18.463 | -1.405 | -1.135 |
| Null Deviance: 165090 on 142028 df | |  |  |  |  |
| Residual Deviance: 15209 on 142018 df | |  |  |  |  |

Table 2. Results of a logistic regression of an epidemic occurring (total infections >=65) or not (total infections <=5) using contact data processed in factorial combinations of temporal sampling window (TSW), minimum contact duration (MCD), spatial threshold (SpTh), and temporal aggregation (Hourly and Daily). “:” indicates interaction between two variables.

|  |  |  |  |  | 95% CI | |
| --- | --- | --- | --- | --- | --- | --- |
| Variable | Estimate | Hazard Rate | *St. Error* | *z* | *LB* | *UB* |
| TSW | -0.006 | 0.994 | 0.005 | -1.240 | 0.985 | 1.004 |
| SpTh | 0.243 | 1.275 | 0.004 | 66.130 | 1.266 | 1.284 |
| MCD | 0.026 | 1.027 | 0.004 | 7.440 | 1.020 | 1.034 |
| Hourly Aggregation | 0.422 | 1.525 | 0.005 | 84.380 | 1.510 | 1.540 |
| TSW:SpTh | -0.712 | 0.491 | 0.004 | -189.150 | 0.487 | 0.494 |
| TSW:MCD | 0.313 | 1.368 | 0.003 | 96.340 | 1.359 | 1.377 |
| SpTh:MCD | -0.234 | 0.791 | 0.002 | -100.740 | 0.787 | 0.795 |
| SpTh:Hourly Aggregation | -0.039 | 0.962 | 0.005 | -8.280 | 0.953 | 0.971 |
| TSW:Hourly Aggregation | 0.065 | 1.067 | 0.006 | 10.400 | 1.054 | 1.081 |
| MCD:Hourly Aggregation | 0.059 | 1.060 | 0.005 | 11.820 | 1.050 | 1.071 |
| R^2^=0.344 |  |  |  |  |  |  |
| Wald Test = 96717 on 10 df | p<0.001 |  |  |  |  |  |

Table 3. Results of a Cox proportional hazards model of time to peak infection in disease transmission models simulated using contact data processed in factorial combinations of temporal sampling window (TSW), minimum contact duration (MCD), spatial threshold (SpTh), and temporal aggregation (Hourly and Daily)**.** “:” indicates interaction between two variables.
