# Supplementary material for: Transmission on empirical dynamic contact networks is influenced by data processing decisions
Source: Epidemics. Author manuscript; Available in PMC 2019 Jul 8. (PMC6613374; doi:10.1016/j.epidem.2018.08.003)
Supplement: 9 [file NIHMS1526165-supplement-9.zip › S9_Dawson et al.2018_Daily Transmission.docx]

Supporting Information 9: Disease model outputs as a factor of log spectral density for all processing combinations at a daily scale, and effect size comparisons between daily and hourly aggregation.


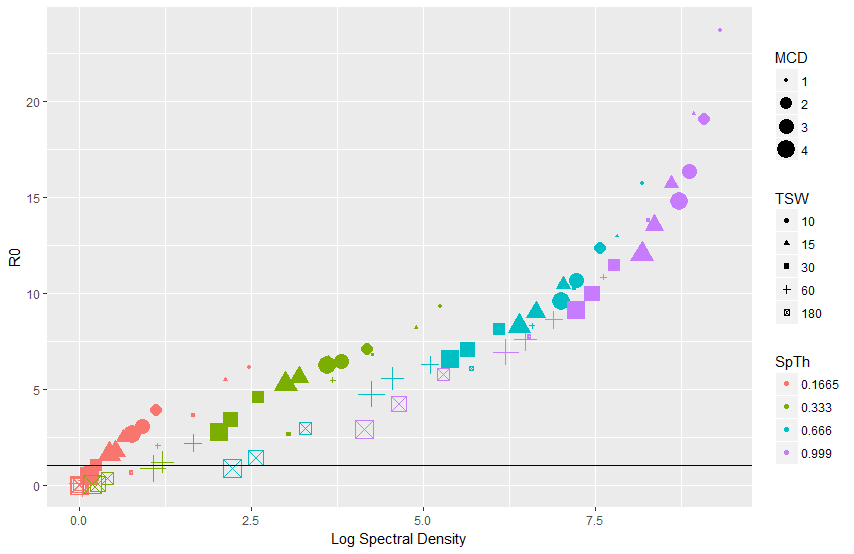


Fig. 1. R_0_ as a factor of the average log spectral density of differently processed datasets aggregated at an hourly basis. SpTh value is indicated by color (orange = 0.1665 m, green = 0.333 m, blue=0.666 m, purple = 0.999 m); TSW is indicated by shape (circle = 10 sec; triangle = 15 sec; square = 30 sec; cross = 60 sec; x-box = 180 sec); MCD value is indicated by size.


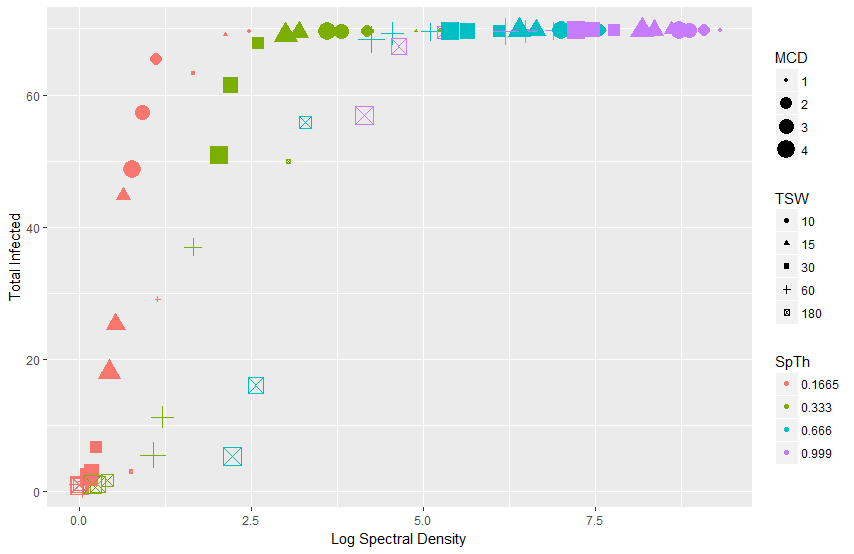


Fig. 2. Total infected individuals as a factor of the average log spectral density of differently processed datasets aggregated at an hourly basis. SpTh value is indicated by color (orange = 0.1665 m, green = 0.333 m, blue=0.666 m, purple = 0.999 m); TSW is indicated by shape (circle = 10 sec; triangle = 15 sec; square = 30 sec; cross = 60 sec; x-box = 180 sec); MCD value is indicated by size.


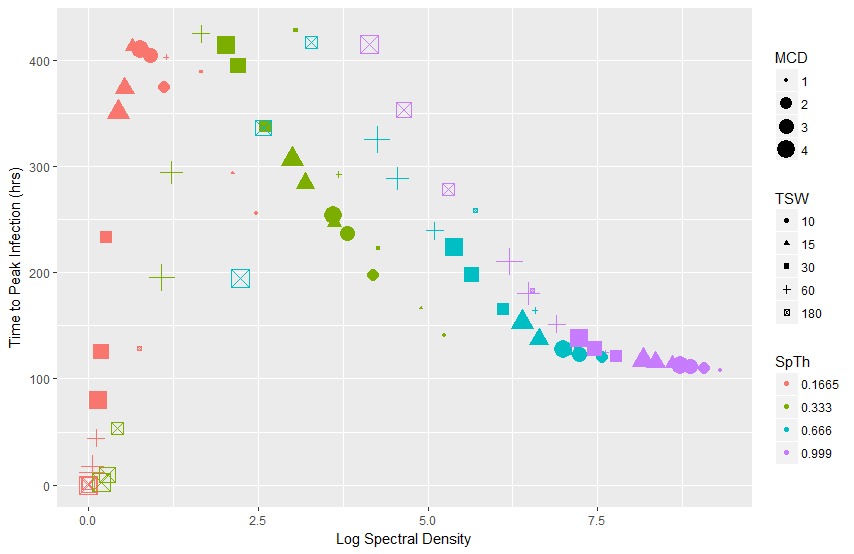


Fig. 3. Peak infection time as a factor of the average log spectral density of differently processed datasets aggregated at an hourly basis. SpTh value is indicated by color (orange = 0.1665 m, green = 0.333 m, blue=0.666 m, purple = 0.999 m); TSW is indicated by shape (circle = 10 sec; triangle = 15 sec; square = 30 sec; cross = 60 sec; x-box = 180 sec); MCD value is indicated by size.

S9.2: Effect size of temporal aggregation

To assess the effect size of temporal aggregation on disease model outputs, effect size (group1_mean_ – group2_mean_/SD_pooled_) was calculated for each factorial combination of SpTh, TSW, and MCD processing parameters. Group mean was calculated as the mean model output for a given factorial combination at either daily or hourly-based aggregation, and SD_pooled_ was calculated as (Nakagawa and Cuthill, 2007):

$$SDpooled= \sqrt{\frac{{\left( n_{2}-1 \right)s}_{2}^{2}+{{(n}_{1}-1)s}_{1}^{2}}{n_{1}+ n_{2}-2}.}$$

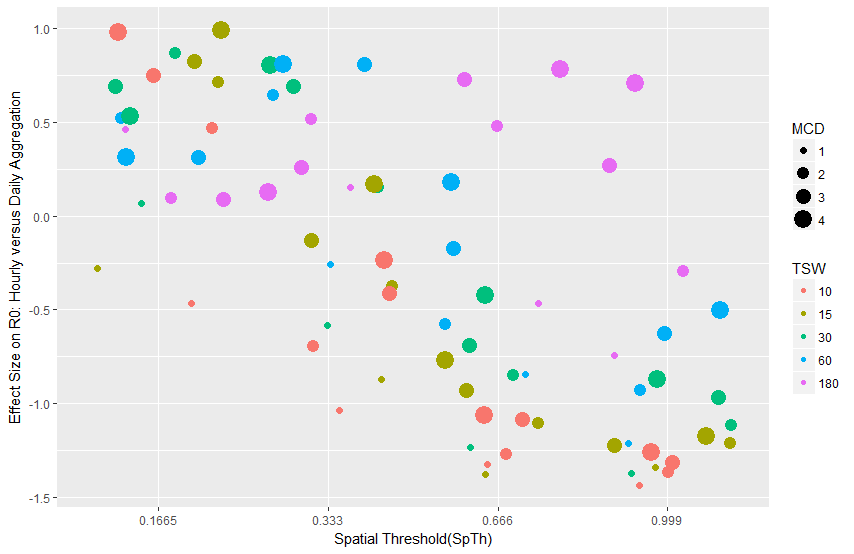


Fig.4. The effect size of temporal aggregation (change in value using hourly versus daily aggregated data) on R_0_ tends to increase with SpTh, and to decrease with TSW and MCD. SpTh is indicated on the x-axis, while MCD is indicated by relative size (small(1) to large (4)), and TSW is indicated by color.


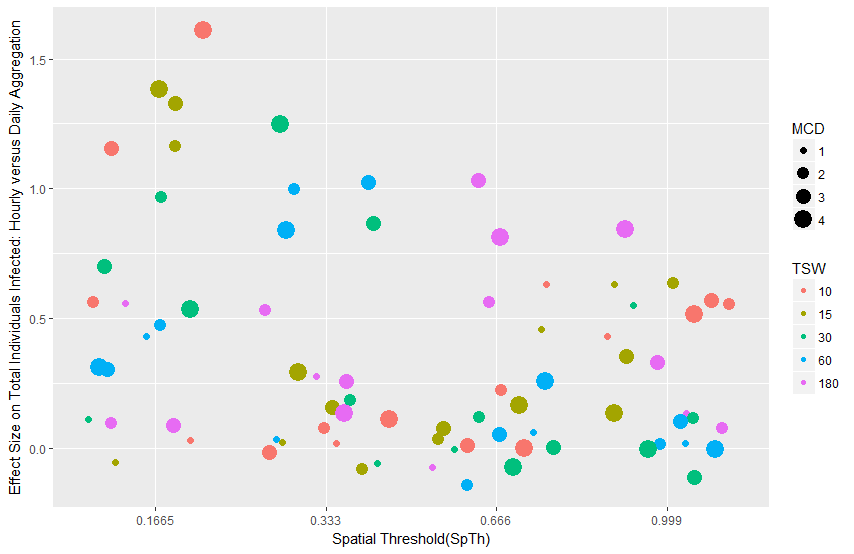


Fig.5.The effect size of temporal aggregation (change in value using hourly versus daily aggregated data) in total number of infected tends to increase with SpTh and TSW, and is largely the same across MCDs. SpTh is indicated on the x-axis, while MCD is indicated by relative size (small(1) to large (4)), and TSW is indicated by color.


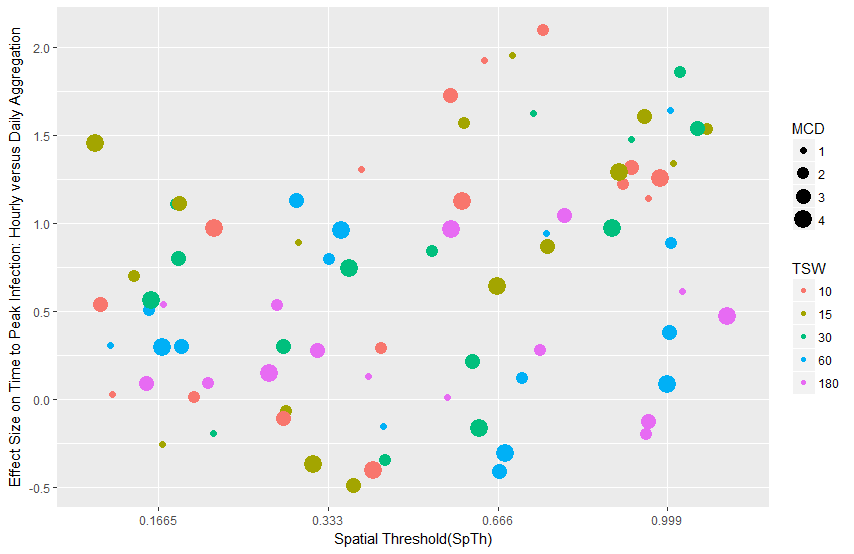


Fig.6. The effect size of temporal aggregation (change in value using hourly versus daily aggregated data) on time to peak infection is variable, and largest for datasets of high relative information (high SpTh & low TSW). SpTh is indicated on the x-axis, while MCD is indicated by relative size (small (1) to large (4)), and TSW is indicated by color.

References:

Nakagawa, S., Cuthill, I.C., 2007. Effect size, confidence interval and statistical significance: A practical guide for biologists. Biol. Rev. 82, 591–605. doi:10.1111/j.1469-185X.2007.00027.x
